# Defective recruitment of motor proteins to autophagic compartments contributes to autophagic failure in aging

**DOI:** 10.1111/acel.12777

**Published:** 2018-05-29

**Authors:** Eloy Bejarano, John W. Murray, Xintao Wang, Olatz Pampliega, David Yin, Bindi Patel, Andrea Yuste, Allan W. Wolkoff, Ana Maria Cuervo

**Affiliations:** ^1^ Department of Developmental and Molecular Biology Albert Einstein College of Medicine Bronx New York; ^2^ Institute for Aging Studies Albert Einstein College of Medicine Bronx New York; ^3^ Marion Bessin Liver Research Center Albert Einstein College of Medicine Bronx New York; ^4^ Department of Anatomy and Structural Biology Albert Einstein College of Medicine Bronx New York; ^5^ Institut des Maladies Neurodégénératives UMR5293 Universite de Bordeaux Bordeaux France; ^6^ CNRS Institut des Maladies Neurodégénératives UMR 5293 C Bordeaux Cedex France; ^7^ Division of Hepatology, Albert Einstein College of Medicine and Montefiore Medical Center Bronx New York

**Keywords:** autophagosomes, autophagy, dynein, intracellular traffic, lysosomes, molecular motors, vesicles

## Abstract

Inability to preserve proteostasis with age contributes to the gradual loss of function that characterizes old organisms. Defective autophagy, a component of the proteostasis network for delivery and degradation of intracellular materials in lysosomes, has been described in multiple old organisms, while a robust autophagy response has been linked to longevity. The molecular mechanisms responsible for defective autophagic function with age remain, for the most part, poorly characterized. In this work, we have identified differences between young and old cells in the intracellular trafficking of the vesicular compartments that participate in autophagy. Failure to reposition autophagosomes and lysosomes toward the perinuclear region with age reduces the efficiency of their fusion and the subsequent degradation of the sequestered cargo. Hepatocytes from old mice display lower association of two microtubule‐based minus‐end‐directed motor proteins, the well‐characterized dynein, and the less‐studied KIFC3, with autophagosomes and lysosomes, respectively. Using genetic approaches to mimic the lower levels of KIFC3 observed in old cells, we confirmed that reduced content of this motor protein in fibroblasts leads to failed lysosomal repositioning and diminished autophagic flux. Our study connects defects in intracellular trafficking with insufficient autophagy in old organisms and identifies motor proteins as a novel target for future interventions aiming at correcting autophagic activity with anti‐aging purposes.

**Abbreviations**
APG: autophagosomes
AUT: autolysosomes
LAMP: lysosome‐associated membrane protein
LC3: light‐chain protein 3
LYS: lysosomes
LysTk: LysoTracker
MVB: multivesicular bodies

## 1 INTRODUCTION

Autophagy is a highly conserved catabolic process responsible for the delivery of cytoplasmic materials (proteins and organelles) into lysosomes for their degradation (Galluzzi et al., 2017; Levine & Klionsky, 2017). Autophagy contributes to maintain cellular and tissue homeostasis by assuring protein and organelle quality control (Kaushik & Cuervo, 2015). A growing number of reports have linked malfunctioning of autophagy with aging, highlighting the role of autophagy as an anti‐aging cellular mechanism (Chang, Kumsta, Hellman, Adams & Hansen, 2017; Cuervo, 2008; Garcia‐Prat et al., 2016; Madeo, Zimmermann, Maiuri & Kroemer, 2015; Rubinsztein, Marino & Kroemer, 2011). Furthermore, genetic inhibition of this degradative process recapitulates features associated with aging and age‐related diseases (Hara et al., 1997; Komatsu et al., 2006, 2007; Menzies et al., 2017). Loss of protein/organelle quality control is a universal hallmark of aging, and malfunctioning of autophagy with age contributes to this gradual accumulation of damaged proteins and dysfunctional organelles (Kaushik & Cuervo, 2015; Kennedy et al., 2014; Lopez‐Otin, Blasco, Partridge, Serrano & Kroemer, 2013). However, the cellular and molecular mechanisms underlying this progressive decline in autophagy during aging remain unknown.

Delivery of cargo (material to be degraded) to lysosomes via macroautophagy, the most conserved and best characterized type of autophagy (hereafter denoted as autophagy), requires regulated trafficking of autophagic vesicles (AVs), the compartments where cargo is sequestered, for their fusion with lysosomes (Galluzzi et al., 2017; Levine & Klionsky, 2017). Subcellular positioning of organelles is mainly determined by the microtubule network and the localization of autophagic and lysosomal compartments is not an exception (Mackeh, Perdiz, Lorin, Codogno & Pous, 2013; Monastyrska, Rieter, Klionsky & Reggiori, 2009). Interaction of these vesicles with microtubules is mediated by motor proteins that provide the force necessary to move them along the tubulin tracks. Vesicle‐associated motors are of two types depending on the direction in which the vesicle is transported: plus‐end‐directed motor proteins (*N‐kinesins*) that transport vesicles toward the cellular periphery and minus‐end‐directed motor proteins (dynein and members of the *C‐kinesin* family such as KIFC2 and KIFC3) that move vesicles to the perinuclear area (Hirokawa, Noda, Tanaka & Niwa, 2009).

The balance between active plus‐end‐ and minus‐end‐directed motors bound to a vesicle's surface determines the directionality of its intracellular movement. In the case of autophagy, the balance of active motor proteins on the surface of autophagosomes has been proposed to prevent their premature or random fusion with lysosomes (Mackeh et al., 2013). In most cells, autophagosome–lysosome fusion occurs mainly in the perinuclear region where it is facilitated through both physical proximity of the organelle and slowing of vesicular trafficking (Kimura, Noda & Yoshimori, 2008; Zaarur et al., 2014). Consequently, efficient positioning of these degradative compartments in the vicinity of the nucleus in a microtubule‐dependent manner is an essential step for the final completion of the autophagic process (Kimura et al., 2008; Monastyrska et al., 2009; Sakai, Araki & Ogawa, 1989). Several reports have shown that the minus‐end‐directed motor protein dynein mediates the centripetal trafficking of autophagosomes (Jahreiss, Menzies & Rubinsztein, 2008; Kimura et al., 2008), but whether or not other minus‐end‐directed motor proteins contribute to the perinuclear location of autophagosomes and lysosomes is unknown.

Autophagic flux (combination of autophagosome biogenesis and their degradation by lysosomes) decreases in an age‐dependent manner in organs such as liver (Bergamini & Kovacs, 1990; Donati et al., 2001). Induction of autophagosome formation in response to liver‐specific stimuli such as glucagon decreases with age (Bergamini & Kovacs, 1990), but despite reduced biogenesis the overall number of autophagosomes is higher in old livers (Stupina, Terman, Kvitmitskaia‐Ryzhova, Mezhiborkaia & Zherebitskii, 1994) due to compromised maturation of autophagosomes into autolysosomes with age (Terman, 1995). Maturation issues are not due to deficiencies in the content of lysosomal enzymes, suggesting that reduced autophagosome clearance with age may be a result of defects in other steps of the autophagic process. Here, we show that defective association with minus‐end‐directed motor proteins by autophagic compartments, specifically autophagosomes and lysosomes, might be a driver of the decline of autophagy with age. Interestingly, the motor defect seems to preferentially affect basal quality control autophagy whereas induction of autophagy by starvation restores in part association of specific motor proteins with autophagosomes and lysosomes. These findings highlight the feasibility of activating inducible autophagy in old organisms to compensate for their defective basal autophagy.

## 2 RESULTS

### 2.1 Differential changes in basal and inducible autophagy with age

**Figure 1:**
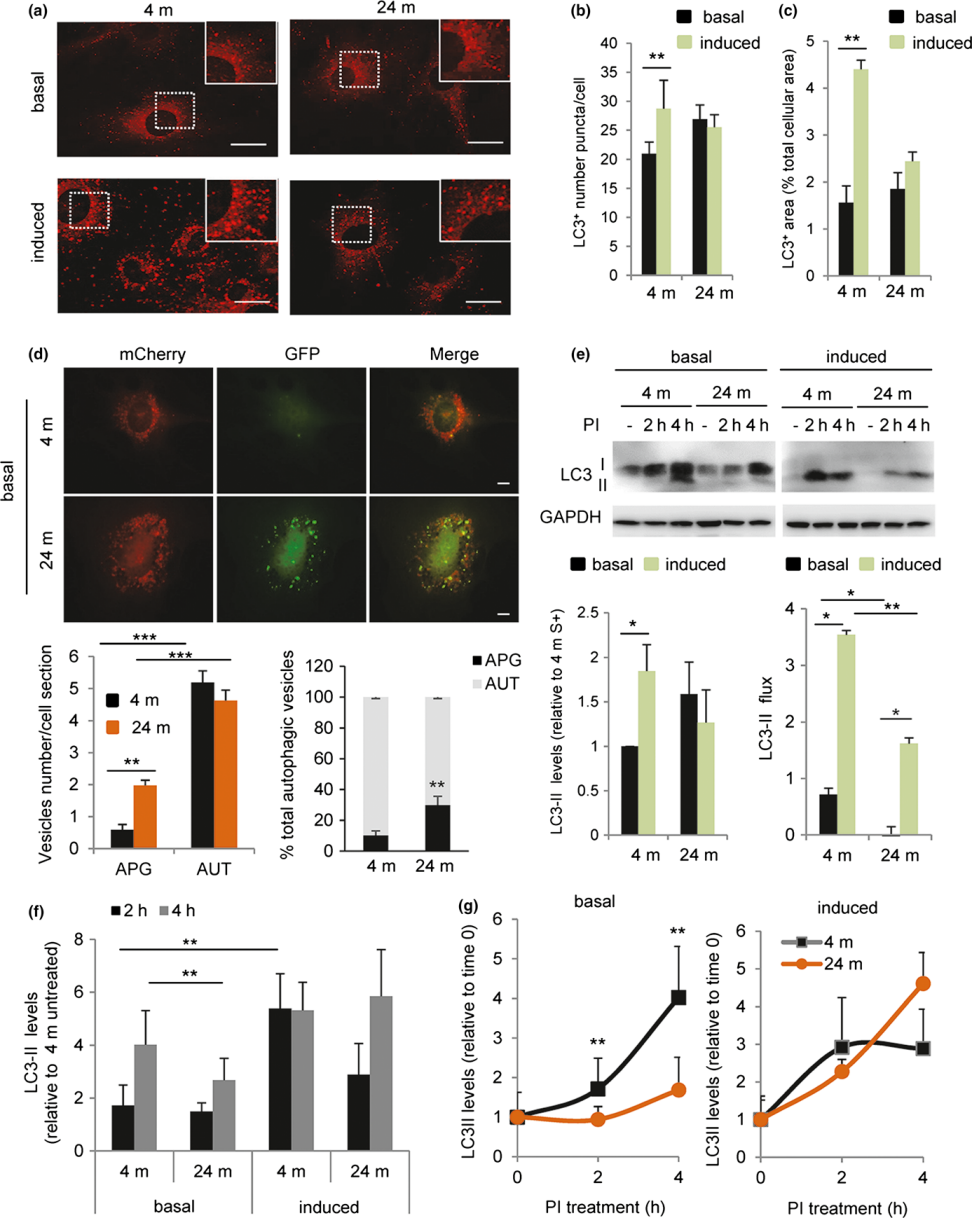
Reduced basal and inducible autophagy in old mouse cells. Primary fibroblasts derived from 4‐m and 24‐m‐old mice were maintained in either presence of serum (basal autophagy) or absence of serum for 4 hrs (induced autophagy). (a) Representative images of immunofluorescence for LC3. Insets show higher magnification. Bar: 10 μm. (b–c) Quantification of (b) number per cell and (c) fraction of cellular area occupied by LC3‐positive puncta (*n* = 4 and >25 cells per experiment). (d) Primary fibroblasts from 4‐m and 24‐m‐old mice were transfected with the tandem reporter mCherry‐GFP‐LC3. Top: representative images of single and merged channels. Bottom: quantification of the number of autophagosomes (APG; mCherry+ GFP+ vesicles) and autolysosomes (AUT; mCherry+ GFP‐ vesicles) (left) and percentage of APG and AUT (right) (*n* = 4 and >25 cells per experiment). (e) Analysis of LC3 flux in cells maintained in presence/absence of serum without additions (−) or in the presence of lysosomal protease inhibitors at saturating concentrations (PI; 20 mm NH4Cl/100 μm leupeptin) for the indicated hours. Top: representative immunoblot. Bottom: quantification of steady‐state LC3‐II levels (left) and LC3‐II flux (right) (*n* = 4). (f) Quantification of the rate of APG biogenesis in experiments as the one shown in E by the difference in LC3‐II levels at 2 and 4 hr after treatment with PI (*n* = 4). (g) Comparison of the changes in LC3‐II levels at different times of addition of PI in cells maintained in the presence (+) or absence (−) of serum (*n* = 4). All values are mean ± *SEM*. One‐way analysis of variance and Bonferroni post hoc test (for multiple comparisons) were used. Differences between 4 and 24 m are significant for *p < .05, **p < .01 and ***p < .001. Absence of symbols indicates no significative difference

We isolated primary skin fibroblasts from 4‐ and 24‐month (m)‐old mice to compare the status of their autophagic system. The use of primary cells avoids possible artifacts of passage in culture and allows analyzing aging separately from senescence. In fact, we confirmed that the isolated skin fibroblasts did not have general senescence features, as positivity for beta‐galactosidase or levels of common senescence marker proteins were comparable in fibroblasts isolated from 4‐m and 24‐m‐old mice (Figure S1). Using these cells, we first analyzed abundance and distribution of autophagic compartments and lysosomes. Labeling for LC3 revealed a comparable number of autophagic vesicles (LC3+ vesicles) in both cell groups under basal conditions (Figure 1a,b). The number of LC3+ vesicles increased significantly in primary fibroblasts from young animals upon 4 hrs of serum removal (induced conditions), a well‐established stimulus of autophagy in cultured cells, but this response was blunted in fibroblasts from old mice (Figure 1a,b). Differences in induced autophagy between primary fibroblasts from young and old animals were even more noticeable when expressed as percentage of cellular area occupied by LC3+ vesicles, to correct for differences in cell size with age (Figure 1c).

Before concluding that differences between both age groups were limited to induced autophagy, we further characterized the degree of maturation of the autophagic compartments under basal conditions. Since LC3 staining cannot differentiate between autophagic vacuoles at different states of maturation, we next used a tandem fluorescent LC3 construct (mCherry‐GFP‐LC3) that allows identifying autophagosomes (APG) as vesicles positive for both fluorophores and autolysosomes (AUT) as vesicles positive only for mCherry (since GFP fluorescence is quenched by the acid lysosomal pH once autophagosomes fuse with lysosomes). Cells from old mice displayed a significantly higher overall content of autophagosomes (Figure 1d), but no increase in overall content of autophagic vacuoles, suggesting possible compromised autophagosome maturation in fibroblasts from old animals even under basal conditions.

Inefficient autophagosome maturation under basal conditions in fibroblasts from the older mice was also confirmed using LC3‐flux assays. Immunoblot analysis for endogenous LC3, to differentiate vesicle‐associated LC3 (LC3‐II) from its cytosolic free form (LC3‐I), confirmed a trend toward higher steady‐state levels of LC3‐II in fibroblasts from old mice and a reduced ability to further increase LC3‐II content in response to starvation in the old group (Figure 1e). Addition of lysosomal protease inhibitors to estimate autophagic flux (as the changes in LC3‐II content after blocking lysosomal degradation) demonstrated that the higher content of LC3‐II under basal conditions in primary fibroblasts from old mice was mostly due to their reduced rates of clearance by lysosomes, because basal autophagic flux was significantly reduced in these cells (Figure 1e). Note that the concentration of protease inhibitors was optimized to assure that all studies were done at a saturating dose for LC3‐II accumulation (data not shown). The defect in basal autophagy in primary fibroblasts from old animals was partially overcome upon serum removal (induced autophagy), but the overall autophagic flux was still significantly lower in the cells from old mice (Figure 1e). These results suggest that both basal and inducible autophagy decrease with age but to a different extent.

Autophagic flux is a combination of both autophagosome formation and clearance. To separately analyze autophagosome biogenesis, we next compared changes in the levels of LC3‐II at 2 and 4 hrs after inhibitor treatment (since degradation is already blocked, an increase in LC3‐II can only originate from de novo synthesis of autophagosomes; Pampliega et al., 2013). These studies revealed significantly lower rates of autophagosome formation under basal conditions in primary fibroblasts from old mice (Figure 1e,f). Reduced autophagosome biogenesis along with the reduced LC3‐II degradation (Figure 1e) contributes to the lower autophagic flux observed in old animals' fibroblasts and explains why despite markedly reduced autophagosome degradation, these cells do not fill up with these vesicles. Interestingly, reduced autophagosome synthesis in fibroblasts from old mice was no longer evident upon serum removal (Figure 1f). In fact, when comparing the dynamics of changes in LC3‐II independently of their starting steady‐state levels, it is easier to appreciate a more pronounced impairment in autophagosome biogenesis in basal autophagy (Figure 1g).

Overall, these findings suggest that basal autophagy and inducible autophagy are affected differently by aging and that reduced autophagy in old organisms is not only due to defective induction (biogenesis), but also to abnormal dynamics of autophagosome clearance/LC3‐II degradation.

### 2.2 Changes in the intracellular distribution of degradative vesicular compartments in aging

Autophagosome clearance is a multistep process that requires trafficking, vesicular fusion, and degradation (Galluzzi et al., 2017; Levine & Klionsky, 2017). Taking advantage of a previously developed cytoskeleton‐free in vitro system that allows directly monitoring of the efficiency of vesicular fusion independent of trafficking (Koga, Kaushik & Cuervo, 2010), we compared the fusogenic properties of autophagosomes (APG) and lysosomes (LYS) isolated from 4‐m and 24‐m‐old mouse livers. We incubated APG from each group with their age‐matched LYS and with LYS from the other age group in a buffer supplemented with ATP, GTP, and Ca^2+^ (all previously shown required for vesicular fusion in this in vitro system;Koga et al., 2010). When both APG and LYS were from aged mice, we found about 30% higher frequency of fusion events than when both vesicles were from young mice (Figure S2a). Interestingly, when old APG were presented to young LYS, fusion frequency was double that seen when both compartments were from young mice (Figure S2a). Separate analysis of the percentage of APG and LYS undergoing fusion revealed that enhanced fusogenicity was mostly driven by the old APG (Figure S2a). These findings suggest that the impairment in autophagosome clearance observed in old cells may not be due primarily to reduced vesicular membrane fusion per se and made us focus our attention on vesicular trafficking instead.

**Figure 2:**
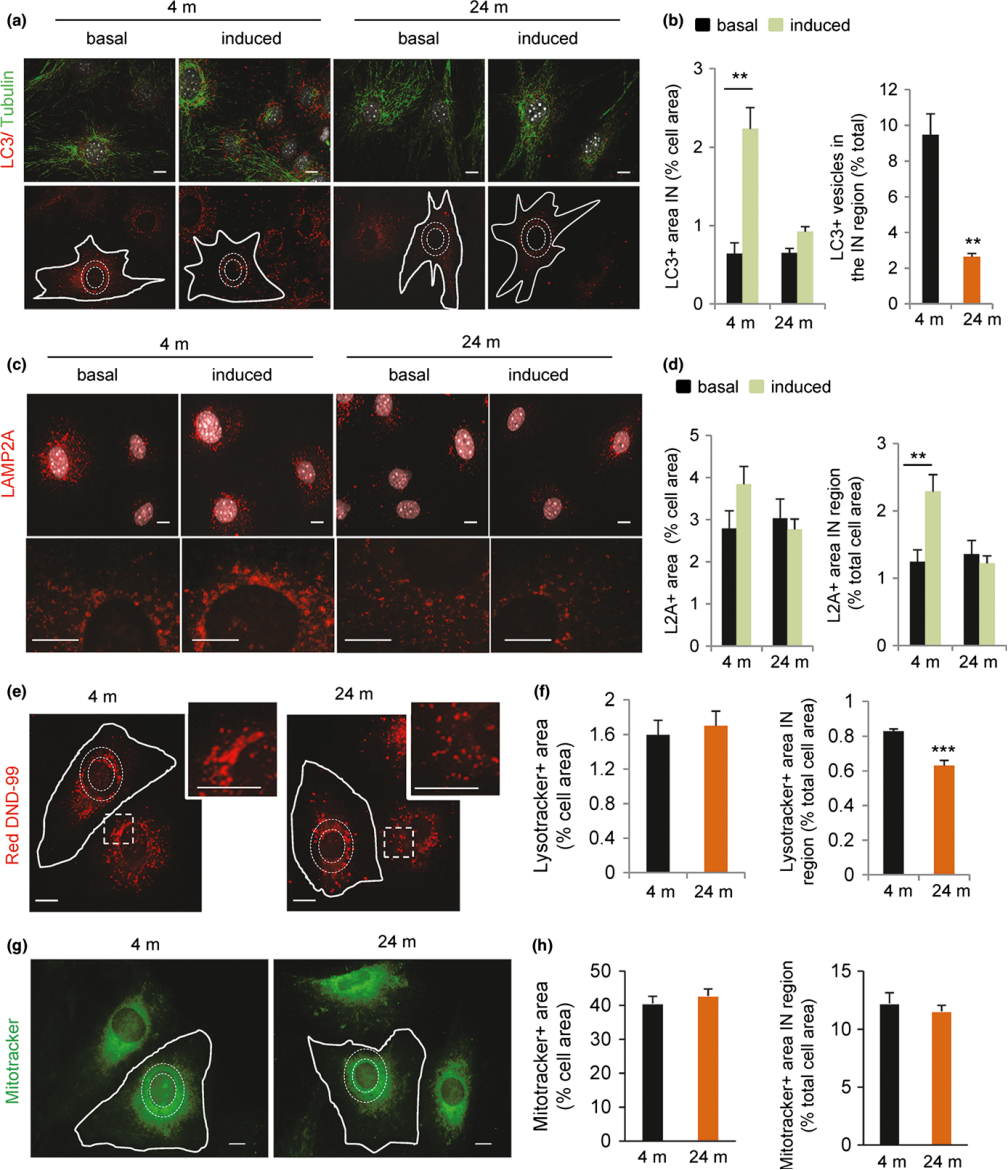
Changes in the intracellular distribution of autophagic compartments in old mouse cells. Primary fibroblasts derived from 4‐m and 24‐m‐old mice were maintained in either presence of serum (basal autophagy) or absence of serum for 4 hrs (induced autophagy). (a) Representative images of immunofluorescence for LC3 (red) and tubulin (green). Nuclei are stained with DAPI (gray). Bottom: Dashed white lines in single red channel indicate the perinuclear region and continuous white lines the cell profile. Bar: 10 μm. (b) Quantification of LC3+ vesicles located in the perinuclear cellular region (IN) from images as the ones shown in a. Values are expressed as the cellular area occupied by LC3+ vesicles (left) or the percentage of LC3+ vesicles located in the perinuclear region in absence of serum (right) (*n* = 4 and >25 cells per experiment). (c) Merged channel image of the staining of the same cells for LAMP‐2A (red) and DAPI (gray). Bottom shows higher magnification regions. (d) Quantification of the fraction of the total cellular area positive for L2A+ vesicles (left) and the fraction of L2A vesicles located in the perinuclear (IN) cellular region (right) (*n* = 4 and >25 cells per experiment). (e–h) Cells were maintained in the absence of serum for 4 hr and incubated with LysoTracker Red DND‐99 (e) or Mitotracker (green, g). Dashed white lines indicate the perinuclear region and continuous white lines the cell profile. Insets show perinuclear regions at higher magnification to illustrate the marked differences in lysosomal density in these regions between fibroblast from 4‐m and 24‐m‐old mice. Bar: 10 μm. Quantification of the total cellular fraction positive for LysoTracker+ vesicles (f left) and the fraction of LysoTracker+ vesicles located in the perinuclear (IN) cellular region (f right) (*n* = 4 and >25 cells per experiment). Same values calculated for Mitotracker+ vesicles (g). Statistical analysis did not reveal significant differences in mitochondrial positioning between both age groups. All values are mean ± *SEM*. Two‐tailed unpaired Student's *t* test (for single comparisons) or one‐way analysis of variance and Bonferroni post hoc test (for multiple comparisons) were applied. Differences between 4 m and 24 m are significant for *p < .05, **p < .01 and ***p < .001. Absence of symbols indicates no significative difference

To start elucidating if differences in vesicular trafficking with age may contribute to the defective autophagic flux, we first analyzed the distribution of LC3+ compartments (autophagic vacuoles, AV) in the cytoplasm of primary fibroblasts from young and old mice. As previously described (Korolchuk et al., 2011), we found that upon removal of serum, the fraction of AVs in the perinuclear region increased about twofold in the fibroblasts from young mice. However, this juxtanuclear relocalization was severely blunted in old mice fibroblasts(Figure 2a,b). We observed similar differences in the subcellular localization of lysosomal compartments labeled with either the lysosome‐associated membrane proteins LAMP2A (Figure 2c,d) and LAMP1 (Figure S2b) or with LysoTracker Red DND‐99 (Figure 2e,f). Although the overall number and area occupied by these compartments were comparable between primary fibroblasts from young and old mice, we found a significant decrease in the number of juxtanuclear lysosomes in the old fibroblasts when compared to young ones (Figure 2d,f). These changes were not seen for all organelles in fibroblasts from old animals as staining of mitochondria did not show significant changes in their distribution relative to the nucleus (Figure 2g,h). Upon transfection of fibroblasts isolated from 4‐m and 24‐m‐old mice with the tandem reporter mCherry‐GPF‐LC3, we found significant reduced juxtanuclear location of AUT in primary fibroblasts from old mice but only a trend in the case of APG, suggesting that the most pronounced changes in vesicular positioning with age occur in the lysosomal compartment (Figure S2c).

These findings support the idea that the juxtanuclear positioning of autophagic vacuoles and lysosomes that occurs during maximal activation of autophagy is impaired in primary fibroblasts from old mice, pointing to the possibility that centripetal motility of the autophagic compartments is deficient with age.

### 2.3 Changes in the motility and directionality of autophagic compartments with age

**Figure 3:**
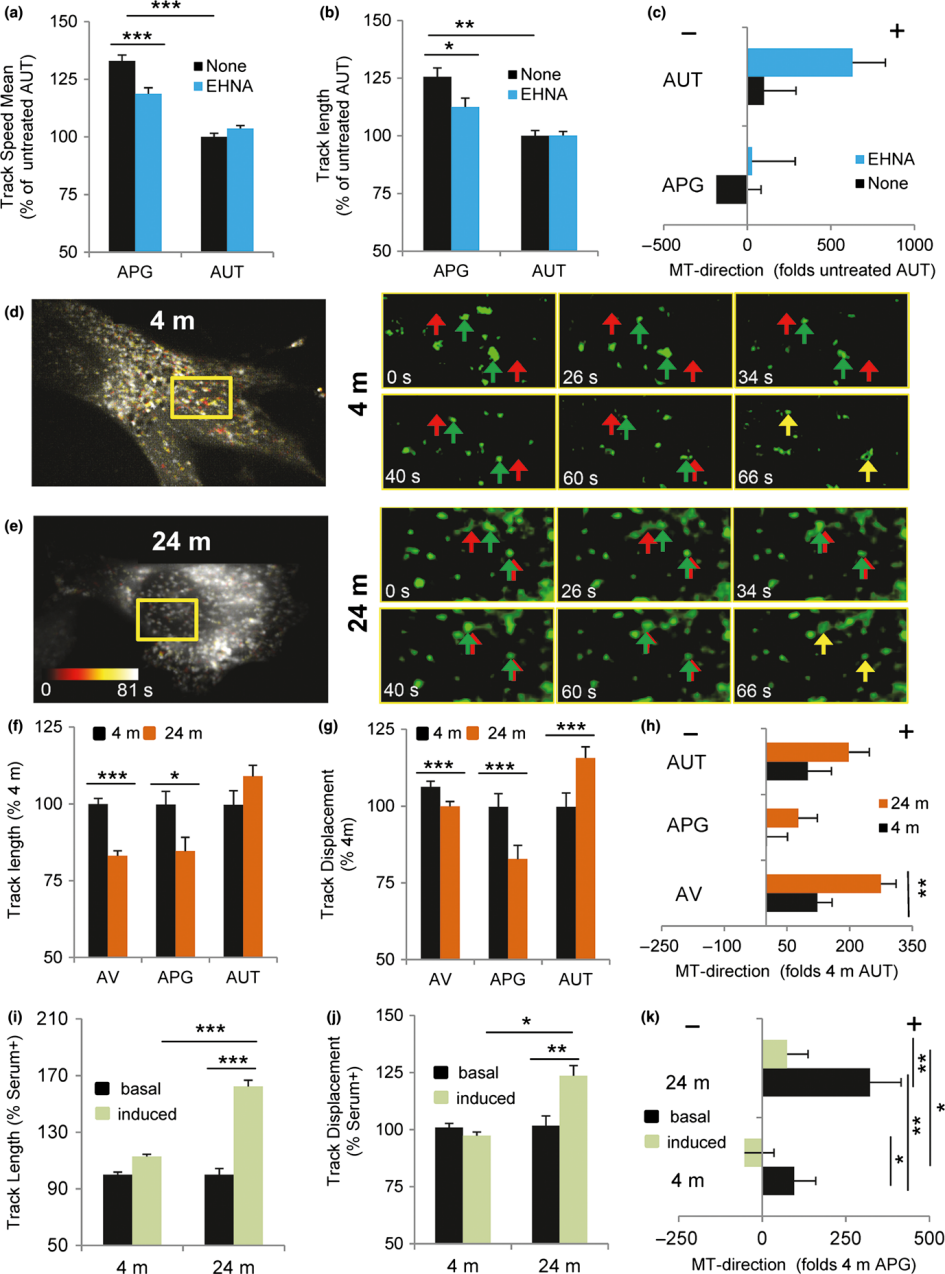
Impact of aging and serum deprivation on the motility properties of autophagic vesicles. (a–c) Analysis of autophagic vesicle motility in primary fibroblasts from 4‐m‐old mice expressing the mCherry‐GFP‐LC3 tandem reporter and maintained in serum supplemented media alone or in the presence of the dynein inhibitor EHNA. Quantification of track mean speed (a), track length (b), and microtubule direction (c), here presented as the net movement per unit time away from (+) or toward (−) the nucleus, with the sign indicating the presumed direction of travel with respect to the microtubule plus or minus‐end of autophagosomes (APG; mCherry+ GFP+ vesicles) and autolysosomes (AUT; mCherry+ GFP‐ vesicles). (d–h) Analysis of vesicular motility in primary fibroblasts derived from 4‐m and 24‐m‐old mice expressing the mCherry‐GFP‐LC3 tandem reporter and maintained in serum supplemented media. Representative cell and sequential frames of GFP‐LC3 at the indicated times of the squared region (length, 19 microns) from 4‐m (d) and 24‐m‐old mice (e). Color scale in the image indicates degree of motility. Left: full field. Right: frames showing example movement of single vesicles (green arrows: current location; red arrows: final location; yellow arrows depict the moment when final and current location coincide (final time)). Quantification of the average of vesicular track length (f), displacement (g), and microtubule direction (h) of autophagic vacuoles (AV; mCherry+ vesicles), autophagosomes (APG; mCherry+ GFP+ vesicles) and autolysosomes (AUT; mCherry+ GFP‐ vesicles). (i–k) Comparison of APG track length (i), displacement (j), and microtubule direction (k) in the same cells but maintained in serum supplemented (+, basal) or depleted (−, induced) media for 4 hr before analysis (*n* = 4 and >25 cells per experiment). All values are mean ± *SEM*. One‐way analysis of variance and Bonferroni post hoc test (for multiple comparisons) were used. Differences are significant for *p < .05, **p < .01 and ***p < .001. Absence of symbols indicates no significative difference

Microtubule‐based motility is required for AV maturation as it mediates relocation of autophagosomes to the proximity of lysosomes (Mackeh et al., 2013). To determine whether inefficient repositioning of AVs with age was due to changes in vesicular motility, we directly analyzed AV dynamics in primary fibroblasts expressing the tandem reporter mCherry‐GFP‐LC3. Using an integrated algorithm for the analysis of continuous single particle tracking, we confirmed in primary fibroblasts from young animals that the dynein inhibitor EHNA (Xu et al., 2014) significantly reduced mean speed and distance covered by APG but did not have any impact on AUT (Figure 3a,b). EHNA treatment accentuated vesicle directionality toward the periphery, in support of a role for dynein in the centripetal movement of AVs (Figure 3c).

Comparative analysis of similar recordings in primary fibroblasts from 4‐m and 24‐m‐old mice (Figure 3d–h) revealed a significant decrease in APG mobility in the older group whereas AUT dynamics were comparable in young and old fibroblasts (Figure 3f,g). In agreement with our steady‐state observations, movement of autophagic vesicles toward the cellular periphery was significantly greater in primary fibroblasts from old mice than from young mice (Figure 3h). Serum removal did not significantly change the overall motility of APG in young cells (Figure 3i,j) but, as previously described (Korolchuk et al., 2011), it switched their microtubule directionality toward the perinuclear area (Figure 3k). Removal of serum in fibroblasts from old animals resulted in a significant increase in APG motility (Figure 3i,j) and partially reverted the directionality of these vesicles (Figure 3k).

These findings highlight again differences between basal and inducible conditions in the impact of aging on autophagic compartments. They also support a positive effect of serum removal in changing vesicle directionality that could explain the beneficial effect observed in autophagic flux in primary fibroblasts from old animals upon serum removal (Figure 1g).

### 2.4 Differences in the recruitment of motor proteins to degradative compartments with age

We next set to investigate the molecular basis of the changes in motility and directionality observed in autophagic compartments with age. Microtubules, motor, and adaptor proteins all participate in AV motility (Mackeh et al., 2013). We did not find obvious differences in the global organization of the microtubule system between young and old primary fibroblasts (Figure S3a,b) or changes in tubulin acetylation (Figure S3c), previously reported to reduce binding of motor proteins to microtubules (Geeraert et al., 2010). Immunoblot analysis of total cellular levels of minus‐end motor proteins, responsible for centripetal vesicular movement, did not reveal significant differences between fibroblasts from young and old mice (Figure S3d).

**Figure 4:**
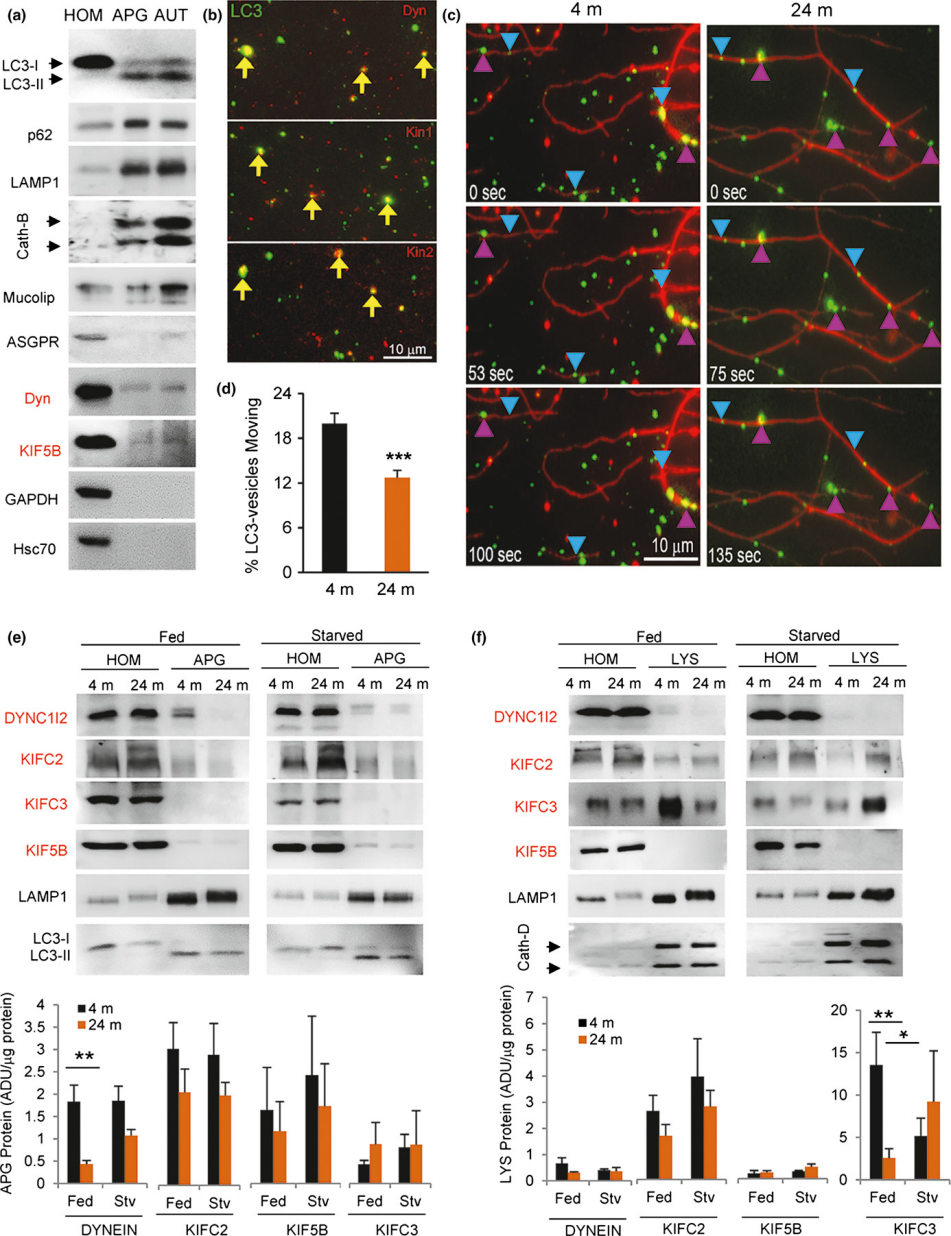
Changes with age in motor proteins associated with autophagic compartments. (a) Immunoblot for the indicated proteins in fractions enriched on autophagosomes (APG) and autolysosomes (AUT) isolated from 4‐m‐old mouse livers. 100 μg of homogenates (HOM) and 20 μg of each fraction were loaded per lane. Molecular motor proteins are labeled in red. (b) Immunostaining of isolated LC3‐positive compartments from 4‐m‐old fibroblasts for the indicated motors (red). Yellow arrows indicate colocalization. (c,d) Analysis of the motility of APG isolated from livers of 4‐m and 24‐m‐old mice in an *in vitro* motility system. Liver vesicles were flowed into a 5‐μl microscopy chamber precoated with Taxol‐stabilized fluorescent microtubules (red), and after binding, these were stained with LC3 antibody (green). (c) Representative fields at the indicated times after addition of ATP. Blue arrowheads indicate examples of moving vesicles, and fuchsia arrowheads indicate examples of nonmoving vesicles. Bar: 10 μm. (d) Percentage of APG (LC3+ vesicles) moving *in vitro* (*n* = 1,328 and 1,267 tracked vesicles). (e,f) Immunoblot for the indicated proteins in APG (e) and LYS (f) isolated from fed or 24 hr starved (Stv) 4‐m or 24‐m‐old mouse livers. 100 μg of homogenates (HOM) and 20 μg of each fraction were loaded per lane. Molecular motor proteins are labeled in red. Bottom: Quantification of the levels of the motor proteins. Values are expressed as arbitrary densitometric units (ADU) per microgram of protein (*n* = 5). All values are mean ± *SEM*. Two‐tailed unpaired Student's *t* test (for single comparisons) or one‐way analysis of variance and Bonferroni post hoc test (for multiple comparisons) were applied. Differences are significant for *p < .05, **p < .01, and ***p < .001. Absence of symbols indicates no significative difference

To directly analyze the content of motor proteins in autophagic compartments, we isolated them from mouse liver. Compromised autophagy with age was initially described in this organ (Bergamini & Kovacs, 1990; Donati et al., 2001) and working with liver allowed us to recover enough organelles for biochemical assays. We isolated APG (enriched in LC3‐II and p62; note that the low amounts of LAMP1 and cathepsin observed in this fraction could represent amphisomes [resulting from fusion of APG and late endosomes]) and AUT (also enriched in both markers but with higher abundance of lysosomal markers (LAMP1, CathB, and mucolipin; Figure 4a ). To determine whether our isolation procedure preserved vesicle‐associated motor proteins, we performed immunoblots for the minus‐end‐directed motor dynein (Jahreiss et al., 2008; Katsumata et al., 2010; Kimura, Noda & Yoshimori, 2007; Kimura et al., 2008) and the plus‐end‐directed motor KIF5B (Cardoso et al., 2009; Geeraert et al., 2010) previously described to contribute to trafficking of autophagic compartments. Immunofluorescence and immunoblot for motor proteins (dynein shown in Figure 4a,b and S4a–c) in the isolated fractions confirmed that motors were indeed present on the surface of the isolated APG and were not due to contamination of the preparation with cytosol. Although present in both fractions, dynein was considerably more abundant in APG than in AUT (note that AUT does not represent the pool of cellular lysosomes, but instead the subpopulation of lysosomes that preferentially fuse with APG).

Similar isolation procedures were performed using livers from 4‐m and 24‐m‐old mice. Using a well‐validated *in vitro* vesicular motility assay (Murray & Wolkoff, 2007), we confirmed that the differences observed in AV motility between in intact cells were still present in isolated organelles. In this cell‐free assay, fluorescent‐labeled isolated organelles are allowed to bind to immobilized purified fluorescent microtubules in microchambers, and the presence of endogenous motor proteins bound to the organelles allows their movement along microtubules upon addition of ATP (Murray & Wolkoff, 2007). As in the case of intact cells, we found that LC3 labeled APG isolated from 24‐m‐old mice were less motile compared to APG isolated from 4‐m‐old mice (Figure 4c,d, Videos S1 and S2 ).

Immunoblot of the isolated fractions revealed that association of dynein is highly reduced in APG from livers of old mice under basal conditions (dynein APG enrichment was 1.83 ± 0.36 and 0.43 ± 0.08 in young and old mice, respectively; *p*: .006; Figure 4e). Interestingly, recruitment of dynein to APG from old mice increased during starvation‐induced autophagy to almost eliminate the differences between both age groups (dynein APG enrichment was 1.85 ± 0.32 and 1.07 ± 0.13 in starved young and old mice, respectively; Figure 4e). Members of the *C‐kinesin* family responsible for minus‐end‐directed motility were not detected in APG from any of the age groups. We detected comparable levels of the plus‐end‐directed kinesin motor protein KIF5B in APG from both age groups (enrichment 1.64 ± 0.95 and 1.16 ± 0.66 in young and old, respectively) suggesting that aging did not interfere with recruitment of this plus‐end motor to APG (Figure 4e). Similar analysis in isolated lysosomes (pre‐APG fusion) revealed that levels of KIF5B were almost negligible in this subgroup of lysosomes. As previously described, it was possible to detect dynein associated with this lysosomal fraction, albeit at lower abundance than previously reported for the overall pool of intracellular lysosomes and in APG (Figure 4e). KIFC2 and KIFC3 were the most abundant motor proteins in these compartments (Figure 4f; note the difference in scale of KIFC3). For these two motors, the most noticeable difference was a significant decrease in levels of KIFC3 in the lysosomes from the old age group under basal conditions (KIFC3 LYS enrichment was 13.54 ± 3.85 and 2.56 ± 1.12; *p*: .03 in young and old mice, respectively; *p*: .004). As in the case of the changes in dynein in APG, starvation diminished the differences in KIFC3 recruitment between the two age groups (Figure 4f). Note that all values are presented relative to protein concentration in the sample as we did not find significant differences for age or nutritional status in the recovery of the APG, AUT, and LYS markers among fractions (Figure S4d,e).

Overall, these findings support age‐dependent differences in the amount of minus‐end‐directed motor proteins associated with mouse liver APG and LYS that are partially reverted by starvation.

### 2.5 Loss of KIFC3 negatively impacts autophagic activity

**Figure 5:**
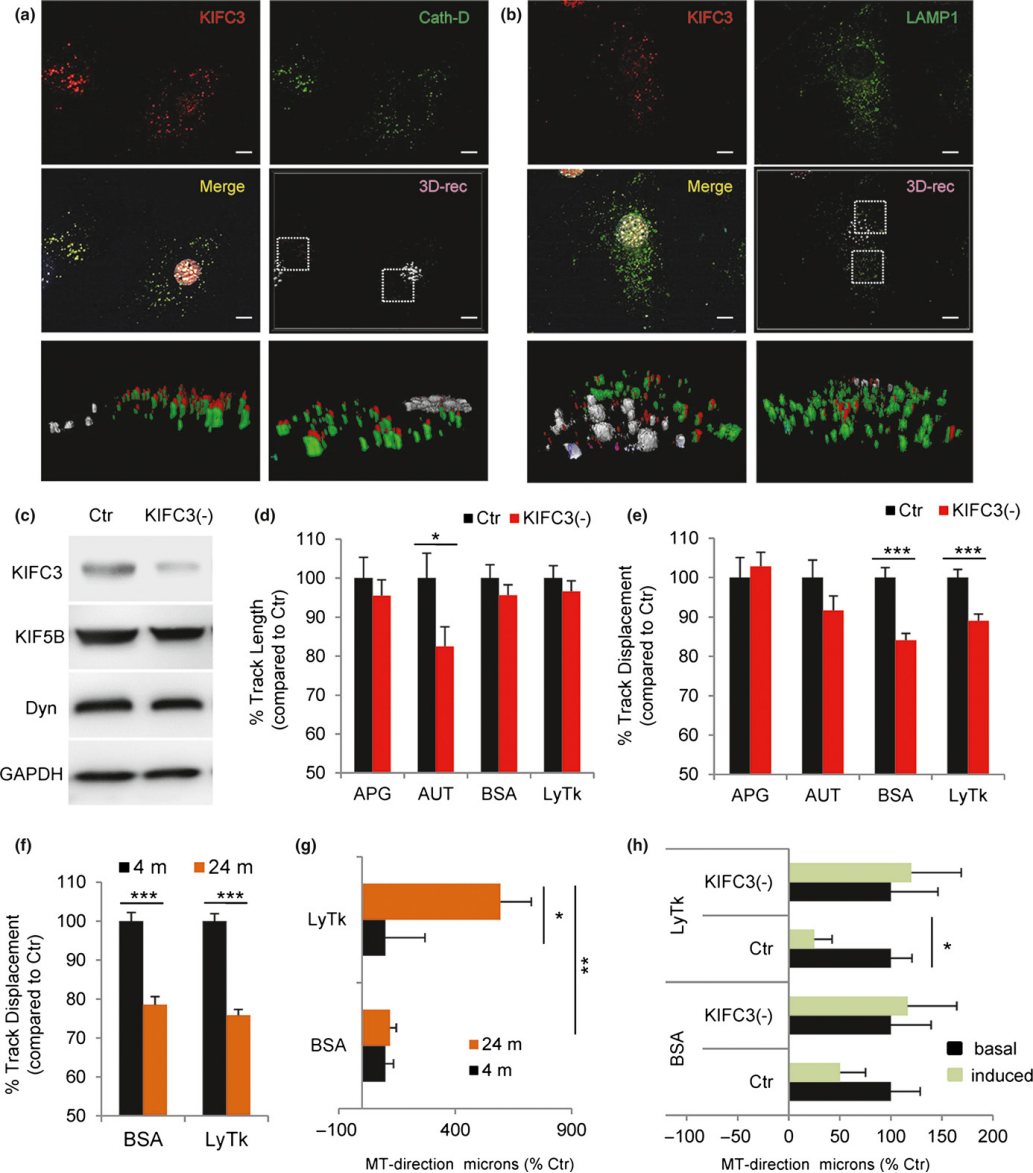
Loss of KIFC3 affects the motility of lysosomal compartment. (a,b) Coimmunostaining for KIFC3 (red) and the lysosomal enzyme Cathepsin D (a) or the endolysosomal marker LAMP1 (b) in primary mouse fibroblasts. Single and merged channels (top) and 3D reconstruction of the boxed regions (bottom). (c) Immunoblot for the indicated proteins of lysates from NIH 3T3 control (Ctr) or knocked‐down for KIFC3 (−). (d,e) Track length (d) and displacement (e) for autophagosomes (APG) (*n* = 438, 638), autolysosomes (AUT) (*n* = 472, 596), BSA (*n* = 1,233, 2,074), or LysoTracker (LysTk) (*n* = 1,835, 2,367) positive vesicles in the same cells stably expressing the mCherry‐GFP‐LC3 tandem reporter or incubated with Alexa647‐BSA or LysTk (*n* = 5 independent experiments). (f,g) Track displacement (f) and microtubule direction (g) of BSA (*n* = 1,887, 1,661) or LysTk (LYS) (*n* = 2,352, 2,494) positive vesicles in primary fibroblasts isolated from 4‐m or 24‐m‐old mice incubated with Alexa647‐BSA or LysTk. (h) Microtubule direction of BSA and LysTk (LYS) positive vesicles in Ctr or KIFC3 (−) fibroblasts maintained in serum supplemented (+, basal) or serum‐free media (−, induced) for 4 hr and incubated with Alexa647‐BSA or LysTk (*n* = 2,150, 2,367, 2,269, 1,835, 1,800, 2,017, 1,324, 1,233 from top to bottom). All values are mean±*SEM*. Two‐tailed unpaired Student's *t* test (for single comparisons) or one‐way analysis of variance and Bonferroni post hoc test (for multiple comparisons) were applied. Differences are significant for *p < .05, **p < .01 and ***p < .001. Absence of symbols indicates no significative difference

Since the role of the minus‐end‐directed motor dynein in retrograde APG trafficking has been well stablished (Jahreiss et al., 2008) and lysosome‐associated KIFC2 did not significantly change with age (Figure 4e,f) and was almost absent in primary fibroblasts (Figure S3d), we set instead to understand the contribution of KIFC3 (the most ubiquitous member of the *C‐kinesin* family) to the aging‐autophagy phenotype because (i) we found that KIFC3 was highly enriched in purified lysosomes (Figure 4f), (ii) its levels were markedly reduced in this compartment in liver from old mice (Figure 4f), and (iii) starvation, at least partially, restored KIFC3 levels in lysosomes (Figure 4f). Three‐dimensional reconstruction of young primary fibroblasts costained for KIFC3 and cathepsin D (Figure 5a) or LAMP1 (Figure 5b) confirmed that, as was the case in liver, KIFC3 was also associated with lysosomes in fibroblasts.

To determine whether KIFC3 contributes to the centripetal movement of lysosomes, we knocked down this motor protein in NIH3T3 mouse fibroblasts using lentiviral‐delivered shRNA (Figure 5c). NIH3T3 fibroblasts were selected because of the technical difficulties in attaining effective and homogenous knockdown in primary fibroblasts before they reached senescence. NIH3T3 cells are spontaneously immortalized fibroblasts that preserve properties of nonmalignant transformed cells, such as contact inhibition, and that we and other have extensively used for characterization of autophagy and vesicular trafficking before (Bejarano et al., 2014). We found reduced displacement of lysosomal vesicles (labeled either with LysoTracker or with Alexa647‐conjugated BSA internalized by endocytosis) but not of APG (positive for LC3) in KIFC3 (−) cells when compared with control (Figure 5d,e). The lysosomal motility behavior in NIH3T3 upon KIFC3 knockdown was comparable to the one we observed in primary fibroblasts from old mice (Figure 5f,g). Tracking lysosomes labeled with either LysoTracker or conjugated BSA revealed a significant reduction in their displacement in primary fibroblasts from 24‐m‐old mice when compared to 4‐m‐old mice (Figure 5f). Lysosomes from old mouse fibroblasts displayed a higher propensity for movement toward the periphery (Figure 5g; in agreement with the lower perinuclear abundance of lysosomes observed in these cells; Figure 2c–f and S2c) that was also evident in KIFC3 (−) cells (Figure 5h). Analysis of the distribution of the LC3 tandem reporter (mCherry‐GFP‐LC3) confirmed that lack of KIFC3 impacted the perinuclear distribution of AUT in basal conditions but not the location of APG (Figure S5a). As observed in primary fibroblasts from old animals, serum removal abrogated the differences observed in AUT between control and KIFC3 (‐) cells (Figure S5a). These findings support the notion that KIFC3 is required for proper directionality of the lysosomal compartment.

**Figure 6:**
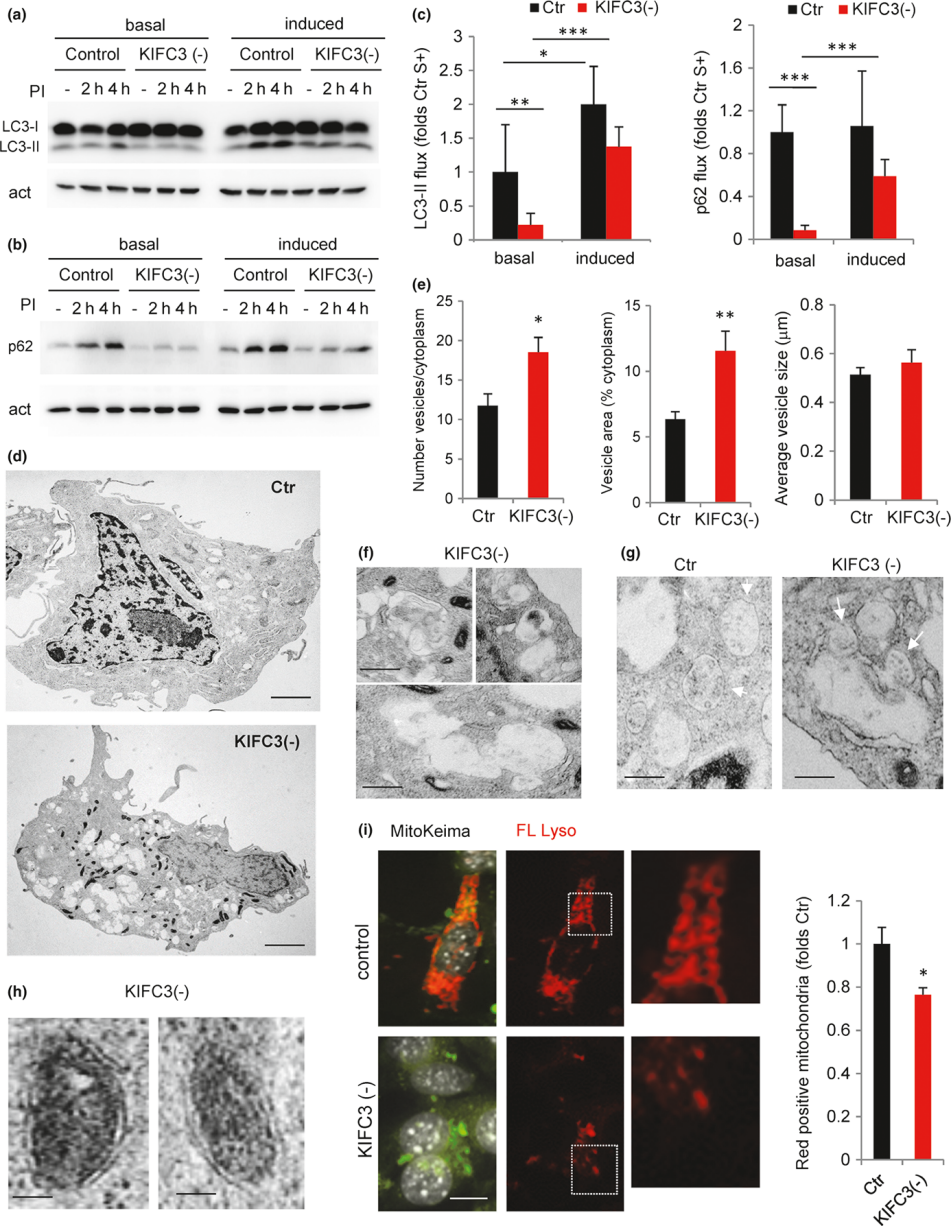
Deficient autophagy upon KIFC3 knockdown. (a–c) Autophagic flux in NIH 3T3 cells control (Ctr) or knocked‐down for KIFC3 (−). Immunoblot for LC3 (a) and p62 (b) in cells maintained in the presence (+, basal) or absence of serum (−, induced) and treated or not (−) with lysosomal protease inhibitors (PI) for the indicated times. (c) Quantification of flux as LC3‐II (left) and p62 (right) degradation after 4 hr. Values are expressed relative to Ctr serum + (*n* = 4). (d–h) Ultrastructure of Ctr and KIFC3 (−) cells. (d) Representative whole‐cell electron micrographs. Bar: 5 μm. (e) Quantification of the number of vesicles (left), percentage of cytoplasm occupied by vesicles (middle), and average size of vesicles (right) in Ctr and KIFC3 (−) cells by morphometric quantification of electron micrographs. (*n* > 10 micrographs). (f) Higher magnification of the vacuolar compartments in KIFC3 (−) cells. Bottom shows several vacuoles with apparently connected lumen, suggestive of homotypic fusion events. Bar: 0.5 μm (g) Examples of multivesicular bodies (MVB) (arrows) in Ctr and KIFC3 (−) cells. Fusion events between MVB and the vacuolar compartments are observed at higher frequency in KIFC3 (−) cells. Bar: 0.5 μm. (h) Higher magnification images of mitochondria sequestered inside double‐membrane vesicles in KIFC3 (−) cells. Bar: 0.5 μm. (i) Ctr and KIFC3 (−) cells transfected with mt‐Keima. Images of merged channels or only red field (FL Lyso). Nuclei are stained with DAPI (gray). Boxed areas are shown at higher magnification to show mitochondria present in acid compartments. Bar: 5 μm right: quantification of the fraction of red‐labeled mitochondria (mitophagy index). Values are shown as fold values in Ctr cells (*n* = 3 and >25 cells per experiment). Two‐tailed unpaired Student's *t* test (for single comparisons) or one‐way analysis of variance and Bonferroni post hoc test (for multiple comparisons) were applied. Differences are significant for *p < .05, **p < .01 and ***p < .001. Absence of symbols indicates no significative difference

Given that genetic suppression of KIFC3 mimicked the dynamics of the lysosomal compartment in primary fibroblasts from old mice, we next analyzed the impact of KIFC3 depletion on autophagy. Immunoblot analysis upon inhibition of lysosomal proteolysis revealed lower degradation rates for LC3‐II and p62 (a well‐characterized autophagic cargo) in KIFC3 (−) cells when compared to control (Figure 6a–c). The lack of p62 accumulation despite its inefficient lysosomal degradation in KIFC3 (−) suggests a possible activation of other nonlysosomal compensatory proteolytic systems in these cells, but future studies are needed to identify the nature of this compensation. Ultrastructure analysis by electron microscopy confirmed a significant increase in the number of vacuolar compartments in the KIFC3 (−) cells (Figure 6d,e). Most of these vacuoles were morphologically compatible with autophagic vacuoles (double or partially double membrane with recognizable cargo inside; Figure 6f) and their abundance in the perinuclear region was in general lower in KIFC3 (−) cells when compared with control (Figure S5b). Interestingly, although serum deprivation was not able to change lysosomal directionality in KIFC3 (−) cells (Figure 5h), this intervention was still able to markedly reduce the differences in autophagic flux between control and KIFC3 (−) cells (Figure 6a,c). These findings suggest that other mechanisms may partially compensate for the deficit in this motor upon serum removal. In that respect, we noticed in KIFC3 (−) cells a higher abundance of late endosome/MVB docking into the vacuolar structures, suggestive of amphisomes that originate from fusion of autophagosomes with late endosomes and have been shown to be increased in conditions with compromised autophagosome/lysosome fusion (Huynh et al., 2007; Figure 6g).

We also noticed that KIFC3 (−) cells displayed a remarkable phenotype of mitochondrial condensation and fragmentation when compared to control cells (Figure 6d and S5c,d). Although it is possible that KIFC3 (−) may also contribute to mitochondrial dynamics, we noticed that many of the condensed mitochondria observed in KIFC3 (−) cells were surrounded by a double membrane, compatible with their being sequestered in undegraded autophagosomes (Figure 6h). To detect possible differences in the ratio of autophagic degradation of mitochondria (mitophagy) between both cell groups, we transfected them with the mitoKeima reporter (Katayama, Kogure, Mizushima, Yoshimori & Miyawaki, 2011), that labels mitochondria in green but it turns red once mitochondria are delivered to acid pH compartments. Compatible with the proposed decrease in mitophagy, KIFC3 (−) cells displayed a marked reduction in the frequency of mitochondria reaching lysosomes (Figure 6i and S5e).

Taken together, these results indicate that KIFC3 acts as the major minus‐end‐directed motor protein required for traffic of lysosomes toward the perinuclear region for their fusion with autophagosomes, and that by reducing levels of KIFC3 to mimic our observations in primary fibroblasts from old animals, it is possible to recapitulate the changes in lysosomal motility and autophagy observed in aging.

## 3 DISCUSSION

The general agreement that failure of the proteostasis network with age contributes to aging (Chang et al., 2017; Cuervo, 2008; Garcia‐Prat et al., 2016; Madeo et al., 2015; Rubinsztein et al., 2011) has called considerable recent attention to autophagy as one of the main components of this network. Despite growing evidence of autophagy dysfunction with age and of the beneficial effects of activating this pathway in experimental models of age‐related disorders (reviewed in (Cuervo, 2008; Rubinsztein et al., 2011; Madeo et al., 2015)), the molecular basis behind autophagic failure in aging requires clarification. In this work, we have found that reduced autophagosome biogenesis and poor autophagosome clearance by lysosomes both contribute to reduced autophagic flux in primary fibroblasts from old mice. We demonstrate that reduced autophagosome clearance is a result of changes in vesicular traffic with age, which we attribute in part, to inefficient recruitment of minus‐end motor proteins to APGs and lysosomes.

Alterations in the last step of autophagy—the degradation of cargo in the lysosomal lumen (Hayasaka, 1989; Ivy, Schottler, Wenzel, Baudry & Lynch, 1984; Nakano, Oenzil, Mizuno & Gotoh, 1995; Tauchi, Hananouchi & Sato, 1980)—and reduced autophagosome/lysosome fusion have both shown to contribute to autophagic failure in the old liver (Terman, 1995). Although the extensive changes in the lipid composition of intracellular membranes reported in old cells (Kitani, 1999; Rottem & Greenberg, 1975) lead to the proposal that reduced vesicle fusogenicity was behind autophagosome maturation, our studies with isolated autophagosomes and lysosomes reveal that fusion between these two compartments is actually enhanced and not reduced. Our study supports the hypothesis that age‐dependent changes in the subcellular distribution of autophagosomes and lysosomes result in reduced autophagosome clearance and that this altered distribution is in part a consequence of changes in trafficking of these organelles.

Changes in the actin and intermediate filament cytoskeletons have been reported in tissues from old organisms including liver (Gourlay, Carpp, Timpson, Winder & Ayscough, 2004; Sun et al., 1998; Tormos et al., 2017). In contrast, few studies have focused on the microtubule cytoskeleton of aging cells, except for the acetylation‐induced instability of neuronal microtubules in the context of dementia (Maxwell et al., 2011; Raes & Remacle, 1987; Schatten, Chakrabarti & Hedrick, 1999). Our microscopic evaluation did not reveal gross changes in the organization of tubulin and acetylated tubulin that could explain the observed changes in autophagic vesicular trafficking. In contrast, our *in vitro* studies in which motility of vesicles prepared from young and old animals was quantified on immobilized exogenous microtubules—support the notion that intrinsic properties of these vesicles underlie the changes in their trafficking with age.

Different factors can contribute to the changes in autophagic vesicle motility with age. It is possible that forming autophagosomes (phagophores) have different motility than fully formed ones and changes in the fraction of phagophores with age could account for the observed differences. However, in previous studies with the LC3‐tandem reporter, we have not observed colocalization of the moving dual color vesicles with phagophore markers such as Atg5 (Bejarano et al., 2014; Pampliega et al., 2013), and consequently, we think that the resolution of our approach is not enough to detect these early forming membranes that contaminate our measurements. Whether differences in the cargo carried by autophagosomes with age could determine their different speed deserves future study. Independent of these possible mechanisms, in this work we have identified a defect at the level of vesicle‐associated molecular motors that can account for the differences in motility with age. Dynein dysfunction with age, manifested as defective interaction with dynactin, has been previously described and shown sufficient to reproduce age‐dependent retromer deficiency (Kimura et al., 2007, 2016). Our analysis of the molecular motors associated with APG and lysosomes in liver confirmed the previously reported presence of the plus‐end‐directed motor KIF5B (member of kinesin‐1 family) in APG (Toda et al., 2008). Although levels of KIF5B in APG did not change with age, we must consider the possibility that changes in motor activity rather than levels may occur in aging. In fact, some of the motor modulators such as the scaffolding protein JIP1 changes kinesin activity in APG but not its vesicular association (Fu, Nirschl & Holzbaur, 2014). However, the dramatic reduction in levels of minus‐end‐directed motors in APG and LYS from old cells is likely the main mechanism by which movement of autophagic compartments is biased toward the periphery in old cells.

Although the large amount of material required prevents us from performing similar biochemical studies in old fibroblasts in culture, the fact that the changes in autophagic flux and in the motility of autophagic vacuoles were comparable in both systems suggest that changes in vesicle‐associated molecular motors could be a common mechanism for defective autophagy in aging. In recent years, an mTORC1‐dependent mechanism has been proposed to modulate the lysosomal positioning during nutritional stress (Korolchuk et al., 2011) but the molecular mechanisms underlying the redistribution of lysosomal compartment remain unknown. Our study suggests that lysosomal association of KIFC3 plays a major role in the retrograde movement of lysosomes and that reduced recruitment of this motor with age contributes, at least partially, to lower autophagy. In fact, the reduced rates of mitophagy observed in KIFC3 depleted cells can explain the abnormal morphology of the mitochondria compartment previously described upon KIFC3 knockdown (Dietrich, Seiler, Essmann & Dodt, 2013). Thus, loss of KIFC3 leads to reduced autophagic activity and perturbed mitochondria morphology/content, two well‐described features of old cells (Kennedy et al., 2014; Lopez‐Otin et al., 2013) .

Also interesting are the differences that we identified in the mechanisms behind failure of basal and inducible autophagy with age. Although both types of autophagy are lower in primary fibroblasts from old mice, defective autophagosome biogenesis is only observed in basal conditions. Starvation seems to restore KIFC3 levels in old lysosomes and thus partially restore defective autophagy in old cells. Changes in motor protein enrichment at the lysosomal membrane could explain the previously described beneficial effect on autophagy in old mice of nutritional interventions, such as caloric restriction (Cavallini, Donati, Gori, Pollera & Bergamini, 2001; Mitchell et al., 2016). In fact, low protein diets alleviate motor symptoms in mice with mutant dynein‐mediated neurodegeneration (Wiesner et al., 2015), suggesting that upregulation of autophagy through nutritional intervention might be beneficial in dynein‐related diseases. It is possible that different mechanisms mediate recruitment of molecular motors to the surface of autophagic compartments under basal and inducible conditions and that those acting on induced autophagy are better preserved with age. For example, higher APG recruitment of dynein during starvation has been linked to post‐transcriptional modifications of tubulin (i.e. acetylation) during autophagic activation (Geeraert et al., 2010) and tubulin acetylation seemed preserved in the old cells used in this study. In addition, several studies have identified changes with age in signaling pathways (i.e. mTOR, SIRT, FOXO) that could contribute to reduced induction of autophagy and autophagosome formation (Bitto et al., 2014; Park et al., 2016; Perluigi, Di Domenico & Butterfield, 2015; Schiavi & Ventura, 2014). Future analysis will be required to explore the molecular requirements that determine the recruitment of motor proteins to AVs and LYS and the potential impact in this process of age‐associated changes in these signaling routes.

Beside dietary interventions, chemical targeting of molecular motors with therapeutic purposes is also gaining momentum. Oncology has been the field that has taken the most advantage of chemical inhibitors of kinesins, now in clinical trials to reduce tumor invasion (Venere et al., 2015). However, other fields such as neurology are following (Lucanus & Yip, 2017). Our findings will call for the use of KIFC3‐activating molecules to compensate for the lower association of KIFC3 in old autophagosomes by increasing their activity. The number of kinesin activators relative to inhibitors is still small, but some small molecules have already been developed and tested (Randall et al., 2017). Even in the absence of a KIFC3 activator, the opposite effect of motor proteins may make it possible to transiently inhibit plus‐end‐directed motor proteins in order to facilitate trafficking and fusion of autophagosomes with the perinuclear lysosomes in old cells. Future studies *in vivo* will also be necessary to evaluate the impact that the microenvironment and intercellular communications that occur in whole tissues may have on KIFC3 recruitment to lysosomes and whether the observed age‐associated differences are tissue‐dependent.

Our study reveals defective vesicular association of molecular motors as a key contributor to reduced autophagy in aging and opens the possibility that correcting this defect, directly or indirectly (through dietary manipulations) may improve vesicular trafficking and consequently restore normal autophagic activity in old organisms.

## 4 EXPERIMENTAL PROCEDURES

### 4.1 Animals, cells, and antibodies

Adult male C57BL/6 mice (4 m and 22–24 m) from the NIA Aged Rodent Colony were used. Where indicated, animals were starved for 6 hrs by completely removing food but maintaining water supply ad libitum. All animal procedures were conducted under an animal study protocol approved by the Einstein IACUC. For the isolation of primary adult fibroblasts, skin biopsies were taken from ears, minced, and incubated in collagenase type II (Gibco, Carlsbad, CA, USA). Primary fibroblasts and mouse fibroblasts (NIH3T3) and IMR‐90 cells were from the American Type Culture Collection. Antibodies sources and dilutions are detailed in Experimental Procedures.

### 4.2 Isolation of subcellular fractions and motility assays

Autophagic compartments were isolated from mouse liver through density gradient centrifugation (Marzella, Ahlberg & Glaumann, 1982). Motility of isolated vesicular fractions was assayed using a microchamber with immobilized microtubules described previously (Murray & Wolkoff, 2007; for details see Experimental Procedures).

### 4.3 Cellular imaging

*Live imaging of p*rimary fibroblasts or NIH 3T3 cells labeled with the indicated organelle trackers was performed using spinning disk confocal microscopy. Particles were tracked from single Z section time series movies with an axial “full width half maximal” thickness estimated at approximately 400 nm for the 1.4 NA, 60× lens. Automated particle tracking using Imaris (Bitplane) software was identical under all conditions, and contribution from Z‐axis movement is not expected to vary. In the case of primary fibroblasts, since the limited number of cell divisions does not allow generation of stable cell lines expressing homogenous levels of the mCherry‐GFP‐LC3 reporter, we selected for quantification analysis cells with similar moderate expression of the reporter to avoid differences due to expression levels. *Indirect immunofluorescence* was performed in cultured cells following conventional procedures (Bejarano et al., 2014) or spotted isolated organelles as described (Koga, Kaushik & Cuervo, 2011; for details, see Experimental Procedures).

### 4.4 Autophagy assays

Autophagic flux was measured by immunoblot for LC3‐II and p62 (Klionsky, 2016) or by imaging with tandem reporter mCherry‐GFP‐LC3 (for in bulk autophagy) or mt‐Keima (Katayama et al., 2011; for mitophagy). Inhibition of lysosomal proteolysis for the flux assays was attained using 20 mm NH_4_Cl and 100 μm leupeptin that we determined were saturating concentrations for blockage of LC3 degradation both in young and in old cells. We opted for this combination as we have extensively shown that at these concentrations and for the duration of our studies (always <6 hr) vesicular fusion was not affected (no additive effect of vinblastine) and that the entire effect can be attributed to reduced degradation at the lysosomal compartment (Klionsky, Elazar, Seglen & Rubinsztein, 2008). In addition, this combination does not have an impact on calcium stores nor does it induce increased autophagosome biogenesis as described for other agents and longer treatments (Alesutan et al., 2015; Rubinsztein et al., 2009). Morphometric analysis of autophagic compartments by electron microscopy was carried out as described previously (Bejarano et al., 2014). *in vitro* fusion of autophagosomes and lysosomes in a microtubule‐free system was measured as described before (Koga et al., 2010; for details, see Experimental Procedures).

### 4.5 Statistical analysis and determination of sample size

All numerical results are reported as the mean ± *SEM* from a minimum of three independent experiments. GraphPad InStat software (GraphPad, La Jolla, CA, USA) was used for analysis of statistical significance. Two‐tailed Student's *t* test for unpaired data was used to evaluate single comparisons between different experimental groups. Differences were considered statistically significant for a value of *p* < .05. For the studies of isolation of lysosomes and cell fractionation, the number of animals for preparation was determined on the basis of the average values of enrichment and recovery for the specific fraction using endogenous markers for each compartment. Power analysis with the sample size utilized and a two‐sided type 1 error rate of 5% predicted >80% power to detect effects of 2.1 or greater in levels of lysosomal components.

## CONFLICT OF INTERESTS

None declared.

## AUTHOR CONTRIBUTIONS

EB designed and performed most of the biochemical and image‐based experiments, analyzed the data, prepared the first draft of the paper, and revised the final version; JM performed *in vitro* and *in vivo* motility analysis and edited the manuscript; BP performed the electron microscopy procedures; OP performed *in vitro* fusion assays; AY assisted with biochemical procedures; AMC and AW conceived and directed the study, contributed to manuscript writing, and edited the final version of the manuscript.
