# Supplementary material for: Defective recruitment of motor proteins to autophagic compartments contributes to autophagic failure in aging
Source: Aging Cell. 2018 May 29;17(4):e12777. doi: 10.1111/acel.12777 (PMC6052466; doi:10.1111/acel.12777)
Supplement: Supplementary file 1 [file ACEL-17-na-s001.pdf]

Defective recruitment of motor proteins to autophagic compartments contributes to autophagic failure in aging

Eloy Bejarano, John W. Murray, Xintao Wang, Olatz Pampliega, David Yin, Bindi Patel, Andrea Yuste, Allan W. Wolkoff and Ana Maria Cuervo

Supplemental Information

Figures S1-5 with legends

Supplementary videos 1 and 2

Extended experimental procedures

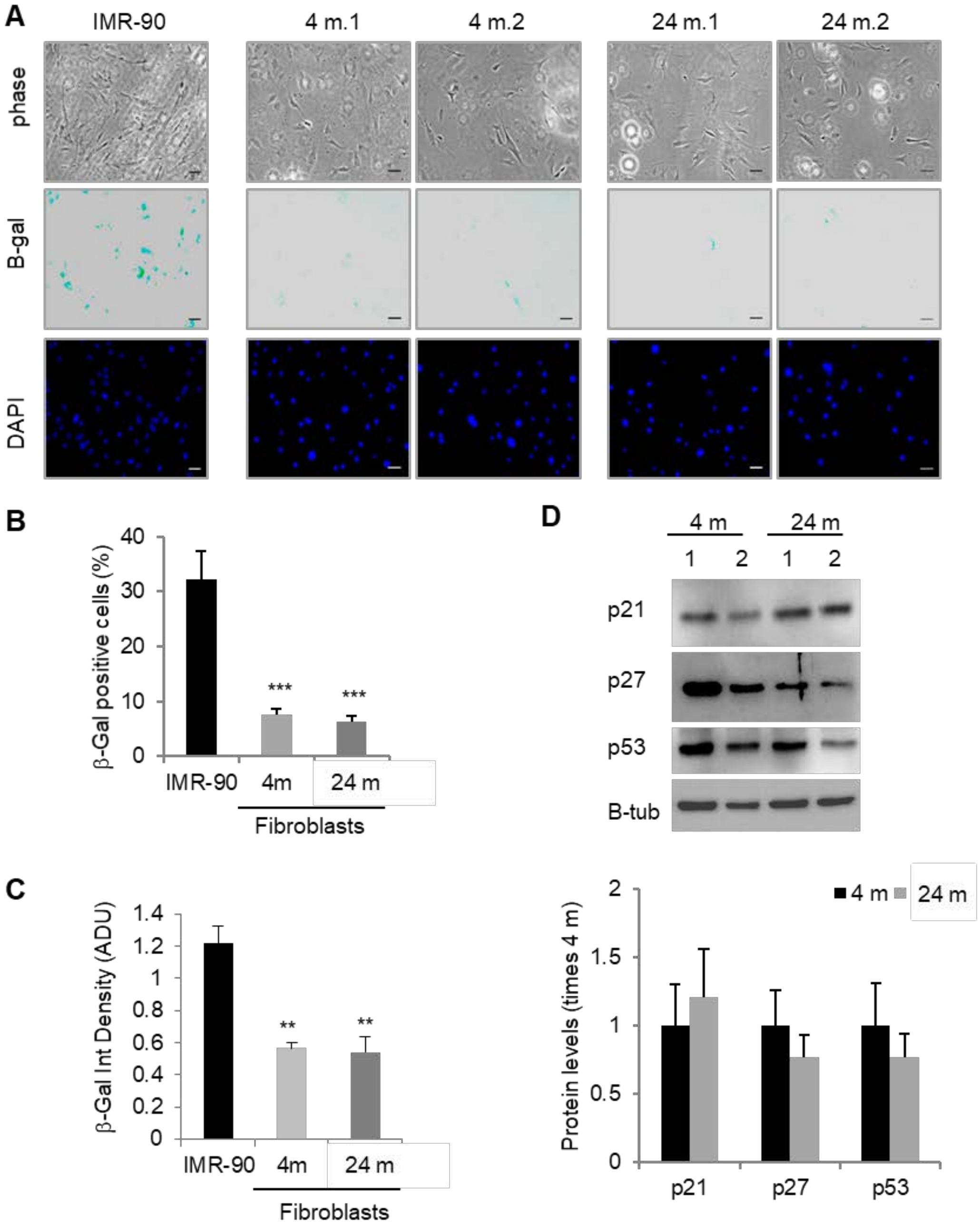

Figure S1. Analysis of senescence markers in primary culture mouse fibroblasts. (**A-C**) β-galactosidase staining in fibroblasts isolated from 4 m and 24 m old mice. (**A**) Representative images of phase, β-galactosidase and DAPI staining. Two independent mice for each age are shown. Senescent IMR-90 fibroblasts at population doubling level (PDL) 50 were used as control for positive staining. Bar: 10 μm. Quantification of percentage of β-galactosidase stained cells (**B**) and intensity of staining normalized to senescent IMR-90 control cells (**C**) (n=4). (**D**) Immunoblot for the indicated senescence markers proteins in the same fibroblasts. Bottom: Quantification of levels of each protein relative to those in 4 m old mice. All values are mean ± s.e.m. Two-tailed unpaired Student's t-test (for single comparisons) was applied. Differences were significant for **$p<0.01$ and ***$p<0.001$. Absence of symbols indicate no significative difference.

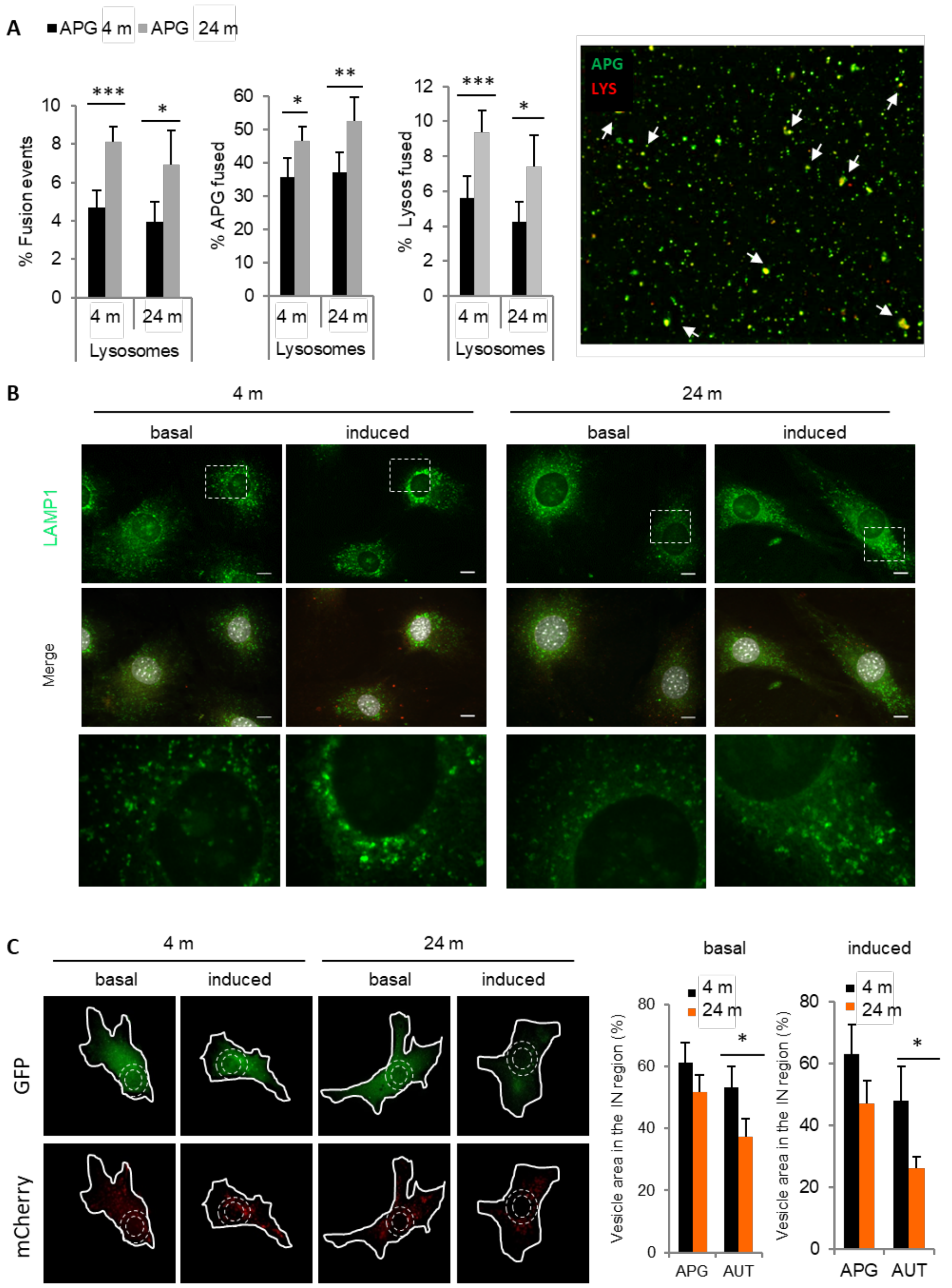

Figure S2. Fusion events and cellular distribution of endo/lysosomal compartments in old mouse cells. (**A**) *In vitro* fusion reconstituted in a microtubule-free system of autophagosomes (APG) labeled in green with anti-LC3 and lysosomes (LYS) labeled in red with anti-LAMP1 isolated from livers of 4 m and 22 m old mice. Percentage of fusion events (left), percentage of APG undergoing fusion (middle), percentage of LYS undergoing fusion (right). n=8-10 fields Right: example of fluorescence image of fusion sample spotted in glass coverslip. Arrows show fusion events. (**B**) Primary fibroblasts derived from 4 m and 24 m old mice were maintained in

presence/absence of serum for 4 hours and immunostained for LAMP1 (green). LAMP1 staining (top), merge with nuclei staining with DAPI (grey) (middle) and higher magnification images of the boxed area (bottom). Bar: 10 μm. (**C**) Primary fibroblasts from 4 m and 24 m old mice were transfected with the tandem reporter mCherry-GFP-LC3 and cultured in presence or absence of serum. Left: representative images of single channels. Dashed white lines indicate the perinuclear region and continuous white lines the cell profile. Right: Quantification of the percentage of the area of autophagosomes (APG; mCherry+ GFP+ vesicles) and autolysosomes (AUT; mCherry+ GFP- vesicles) located in the perinuclear cellular region (IN). All values are mean ± s.e.m. Two-tailed unpaired Student's t-test (for single comparisons) or one-way analysis of variance and Bonferroni post hoc test (for multiple comparisons) were applied. Differences were significant for *$p<0.05$, **$p<0.01$ and ***$p<0.001$. Absence of symbols indicate no significative difference.

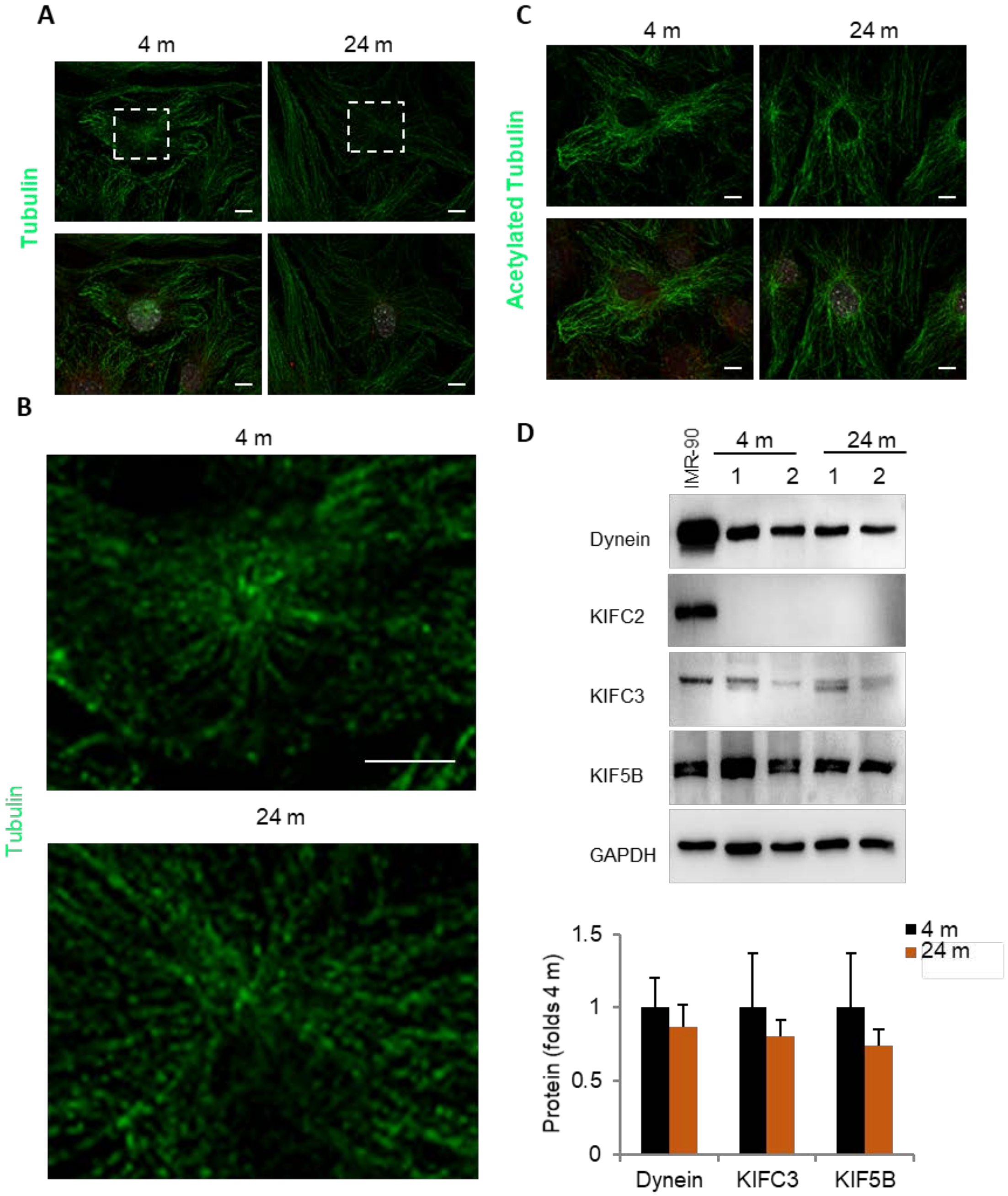

Figure S3. Cytoskeleton, motor proteins and autophagic vacuole fusogenicity in primary fibroblasts from old mice. (**A-C**) Primary fibroblasts derived from 4 m and 24 m old mice immunostained for tubulin (**A, B**) or acetylated tubulin (**C**). DAPI stained nuclei are shown in grey in **A** and **C** Bar: 10 μm. The microtubular organizing centers (boxed areas in A) are shown at higher magnification in **B.** Bar: 2 μm. (**D**) Immunoblot for the indicated molecular motors in total cellular lysates from the same cells as in Fig. S1. Bottom: Quantification of levels of each protein relative to those in 4 m old mice. Values are all mean ± s.e.m. Two-tailed unpaired Student's t-test was used. Absence of symbols indicate no significative difference.

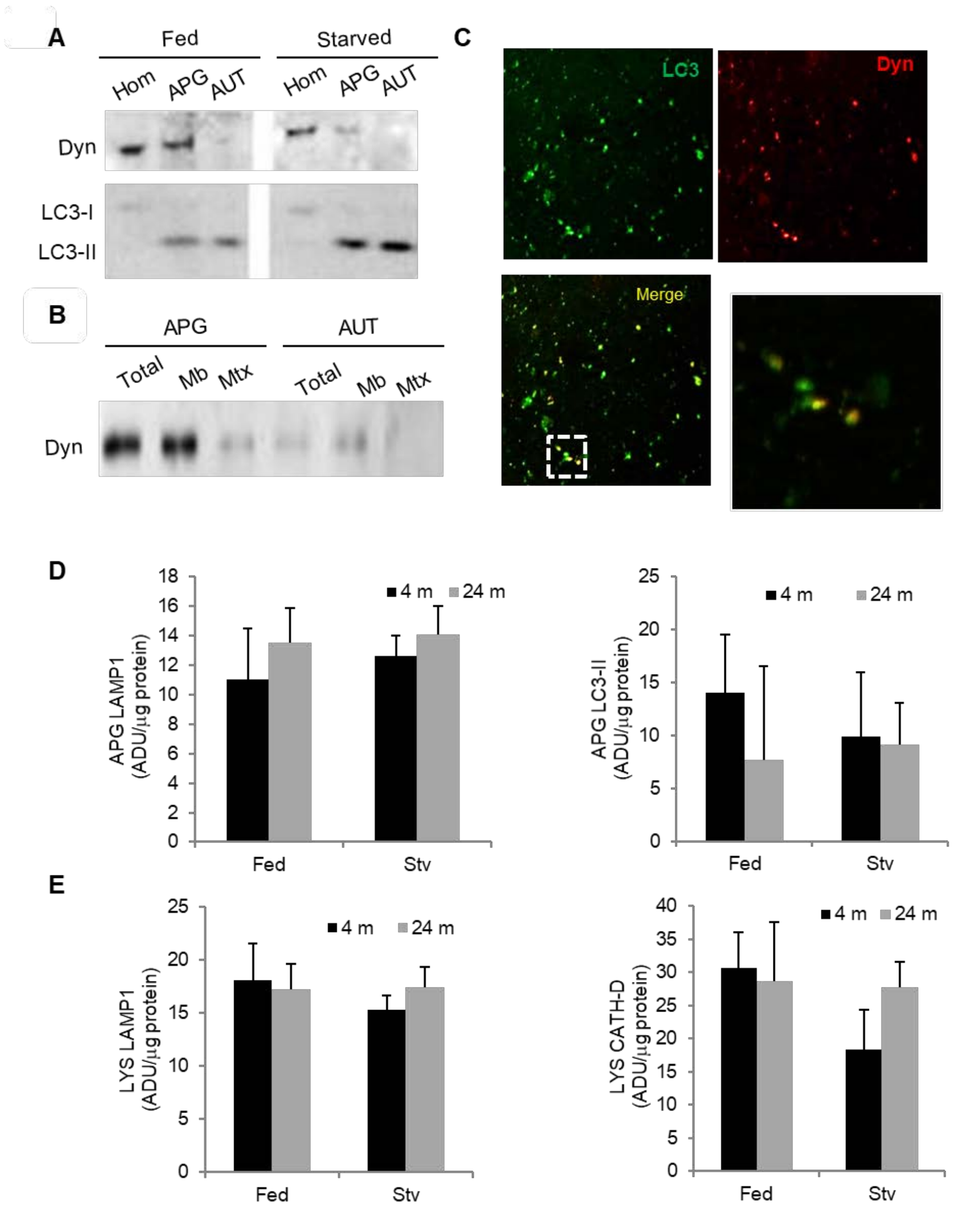

Figure S4. Association of molecular motors to autophagic compartments. A-B. Immunoblot for dynein (Dyn) of autophagosomes (APG) and autolysosomes (AUT) isolated from livers of normally fed or 24h starved mice (**A**) and in membranes (Mb) and matrices (Mtx) of these compartments separated by a hypotonic shock and collected by centrifugation (**B**). Hom: homogenate. (**C**) APG derived from 4 m old mice immunostained for LC3 (green) and dynein (red). Single channels and merged are shown. Boxed area is shown at higher magnification to illustrate colocalization between both markers. (**D, E**) Quantification of immunoblots for the indicated proteins in autophagosomes (APG) (**D**) and lysosomes (LYS) (**E**) isolated from fed or 24h starved (Stv) 4 m or 24 m old mouse livers. Representative immunoblots are shown in main figure 4D, E. Values are expressed as arbitrary densitometric units (ADU) per microgram of protein (n=5). All values are mean ± s.e.m. No significant differences were detected.

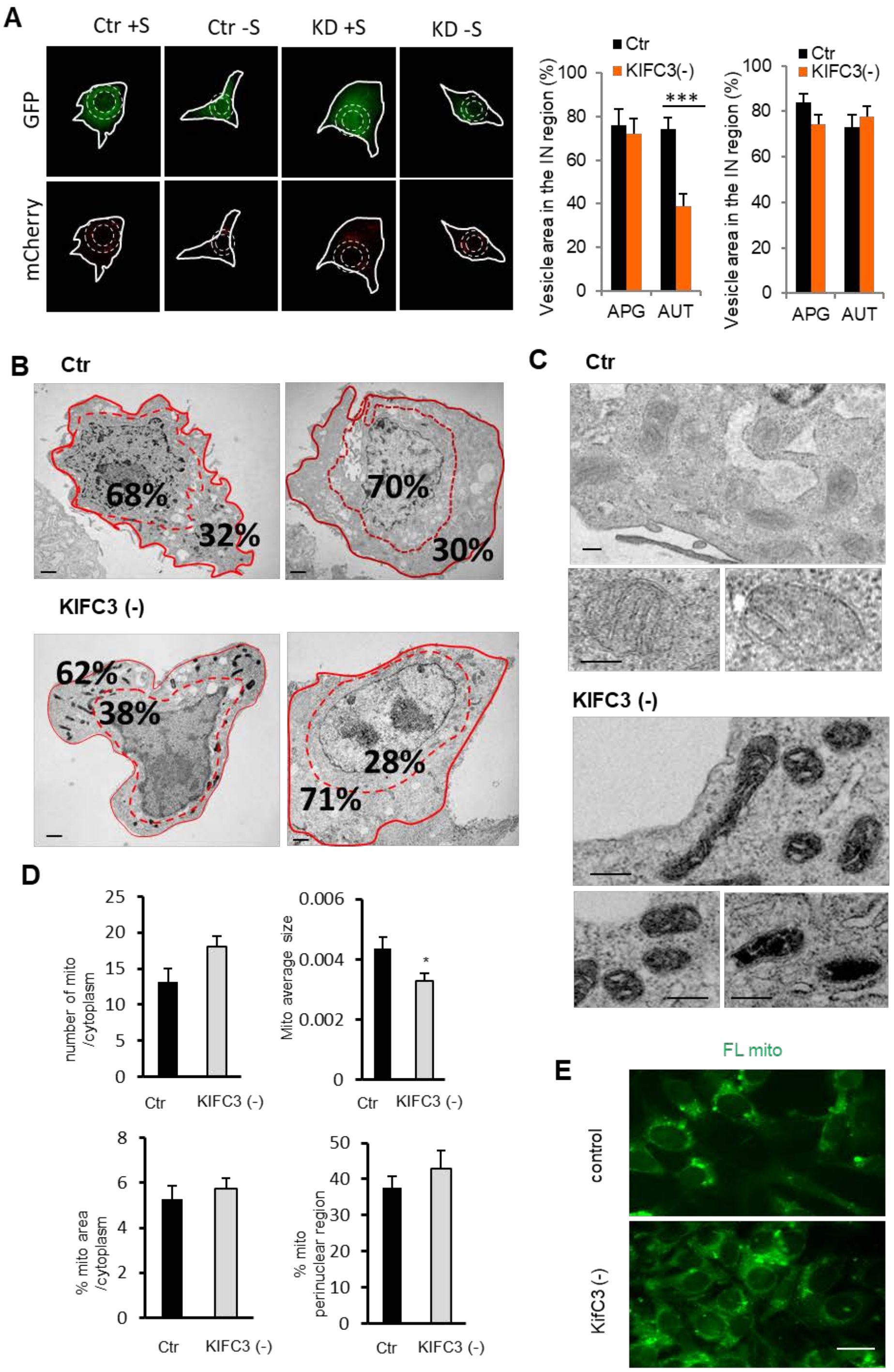

Figure S5. Ultrastructure of NIH 3T3 cells depleted of the motor protein KIFC3. (**A**) NIH 3T3 cells control (Ctr) or knocked-down for KIFC3 (-) were transfected with the tandem reporter mCherry-GFP-LC3 and cultured in presence or absence of serum. Left: representative images of single channels. Dashed white lines indicate the perinuclear region and continuous white lines the cell profile. Right: Quantification of the percentage of the area of autophagosomes (APG; mCherry+ GFP+ vesicles) and autolysosomes (AUT; mCherry+ GFP- vesicles) located in the perinuclear

cellular region (IN). (**B**) Representative micrographs of NIH 3T3 cells control (Ctr) or knocked-down for KIFC3 (-). Example of profile tracing to quantify the fraction of vesicular compartments inside and outside of the perinuclear area as indicated in the images. Continue line tracing: cell periphery. Discontinue line tracing: perinuclear region. Bar: 5 μm. (**C**) Higher magnification images of micrographs of the same cells to show characteristics of the mitochondria morphology in both types of cells. Bar: 0.5 μm. (**D**) Morphometric analysis of electron micrographs from the same cells. Number and average size of mitochondria (top) and percentage of cytosolic area occupied by mitochondria and fraction of mitochondria in the perinuclear region (bottom) (n>10 micrographs). (**E**) Green channel image of Ctr and KIFC3 (-) cells transfected with mt-Keima. Red channel and merge are shown in Fig. 6I. Bar: 10μm. All values are mean ± s.e.m.One-way analysis of variance and Bonferroni post hoc test (for multiple comparisons)was applied. Differences were significant for *p<0.05 and ***p<0.001. Absence of symbols indicate no significative difference.

Supplementary Videos

Supplementary Video 1. Motility of APG isolated from livers of 4 m old mice in an *in vitro* motility system. Liver vesicles were flowed into a 5 µl microscopy chamber pre-coated with Taxol-stabilized fluorescence microtubules (red), and after binding, these were stained with LC3 antibody (green).

Supplementary Video 2. Motility of APG isolated from livers of 24 m old mice in an *in vitro* motility system. Liver vesicles were flowed into a 5 µl microscopy chamber pre-coated with Taxol-stabilized fluorescence microtubules (red), and after binding, these were stained with LC3 antibody (green).

Extended experimental procedures

Cell culture

Cells were cultured in Dulbecco's modified Eagle's medium (Sigma) containing 10 % Fetal bovine serum (FBS), 50 μg ml−1 penicillin, and 50 μg ml−1 streptomycin at 37 °C with 5 % CO2. All cells were tested for mycoplasma contamination using a DNA staining protocol with Hoechst 33258 dye. When indicated, cells were washed three times with Hank's balanced salt solution (HBSS; Invitrogen, Carlsbad, CA) and placed in fresh medium without serum to activate the autophagic process. Where indicated, lysosomal proteolysis was inhibited by addition of 20 mM NH4Cl and 100 μM leupeptin (Fisher Scientific). The β-galactosidase staining kit (Cell Signaling Technology) was used to measure senescence in primary cultures. Senescent IMR-90 cells (population doubling level 50) were used as positive control.

Antibodies

Antibodies were used at dilutions 1:1,000 for immunoblot and 1:200 for immunofluorescence, except where stated differently. The antibody against LAMP2A (#512200) was from Invitrogen; against Hsc70 (#NB1202788) from Novus; against p62 (#BML-PW9860) from Enzo Life Sciences against acetylated tubulin (#T7451), β-tubulin (#T8328) and KIFC3 (#HPA021240) were from Sigma-Aldrich; against GAPDH (#ab8245),actin (#ab6276), p21 (#ab109199), dynein (#ab167399), KIF5B (#ab167429) and kin-2 (#ab109528) from Abcam (Cambridge, MA); against LC3 (#2775), p53(#2524) and p27(#2552) from Cell Signaling Technology (Beverly, MA); against LC3 (#M152-3 and #PM036) from MBL; against Cathepsin D (#sc-6486; 1:250 IB; 1:50 IF) from Santa Cruz (Santa Cruz, CA); against KIFC2 (#PA5-32165) was from Pierce; against LAMP1 (clone 1D4B) from the Developmental Hybridoma Bank (University of Iowa, Iowa City, IA). The antibody against mucolipin was a gift from R.Nixon (New York University, USA). Affinity purified rabbit IgG was prepared against a KLH-linked peptide (VNRWACERKRDITYC) corresponding to a sequence on the cytoplasmic tail of the rat asialoglycoprotein receptor (ASGPR) H2 subunit as described before (Treichel *et al.* 1994). All

secondary antibodies for immunofluorescence were from Molecular Probes (Invitrogen). Formaldehyde and paraformaldehyde were from PerkinElmer Life and Analytical Sciences (Waltham, MA).

Cell transfection and RNA interference.

Cells were transfected with cDNA constructs mt-Keima (Katayama *et al.* 2011) and mCherry–GFP–LC3 (Kimura *et al.* 2007) using Lipofectamine2000 (Invitrogen) according to the manufacturer's instructions. Knock-down was performed using lentiviral-delivered small hairpin RNA (shRNA) from the Mission-Sigma library (Sigma-Aldrich) against KIFC3 (5′-CAACGACTACAATGGGCTCAA-3′) (TRCN0000116466) and transduction with virus carrying the empty vector was used as control. Lentiviral particles were generated by co-transfection with the third-generation packaging constructs pMDLg/pRRE and pRSV–REV, and as envelope the G glycoprotein of the VSV (pMD2.G) into HEK293T cells as described before (Massey *et al.* 2008).

Isolation of subcellular fractions from mouse liver

Autophagosomes (APG), autolysosomes (AUT) and lysosomes were isolated from liver of fed or 6-h-fasted mice by centrifugation in a discontinuous metrizamide density gradient as described previously (Marzella *et al.* 1982). Fractions were recovered from the different metrizamide interfaces (APG in 20–15 %, APL in 20–24 % and lysosomes in the 26–24 %) and washed by centrifugation in 0.25 M sucrose. The purity of the different subcellular fraction was confirmed by biochemical analysis of protein markers for different compartments. For vesicle motility assays, highly concentrated vesicles were obtained by adaptation of previous vesicle preparations (Murray & Wolkoff 2007). Mice were anesthetized with ketamine/xylazine and injected with the fluorescent hepatocyte ligand, Texas red ASOR (50µg), in the liver portal vein. Ligand was allowed to be endocytosed for 5 min, livers were removed and dounce homogenized in MEPS buffer (35 mM K_2-PIPES, 5 mM EGTA, 5 mM $MgCl_2$, 0.25 M sucrose pH 7.4) containing 4 mM DTT, 1:20 protease inhibitor (Sigma Cat. #P-8340), and 15 mM phenylmethanesulfonyl fluoride.

The homogenate was then centrifuged 2500 x g for 10 min, and the post nuclear supernatant was collected, mixed with 80% Nycodenz (Accurate Chemical and Scientific Corp, Westbury, MA) and loaded into the bottom of a Nycodenz step gradient consisting of 50, 24.1, 17.2, and 0 % Nycodenz (w/v) in MEPS. This was centrifuged 200,000 x g for 2 hours. The cloudy layer at each interface was collected and frozen as 15 µl single use aliquots. Vesicles floating above 17.2 % Nycodenz were used for motility assays.

Vesicle Motility Assay

Motility assays were performed in a 3 µl optical chamber flow cells consisting of 24x50 mm coverglass on the bottom, two strips of double-sided tape to from a channel and cut glass to form a cover as described previously (Murray & Wolkoff 2007). Fluorescent microtubules in MT buffer (80 mM K_2-PIPES, 1 mM EGTA, 1mM $MgCl_2$, 3% glycerol, 1mM GTP, pH 7.0) were flowed into chambers coated with 30µg DEAE-dextran to allow their attachment. Chambers were washed and vesicles were flowed in and allowed to bind to the microtubules on ice for 15 min. Chambers were washed and vesicles were then labeled with primary antibody to LC3 and p62, by washed, and visualized by and fluorescent secondary antibody. For image acquisition, chambers were removed from ice and placed on the fluorescence microscope stage maintained at 37°C. Time-lapse image collection was initiated followed by addition of 50 µM ATP, to induce motor dependent vesicle motility. Motility assay buffer for washes consisted of PMEE buffer (35 mM K2 -PIPES, 5 mM MgCl2 , 1 mM EGTA, 0.5 mM EDTA, pH 7.4), plus 2 mg/mL bovine serum albumin, 20 µM Taxol, 4 mM DTT, 2 mg/mL Na-ascorbic acid.

Live cell imaging and particle tracking

Cells were cultured in DMEM medium lacking phenol red on 4-well coverglass bottomed chambers (In Vitro Scientific, Sunnyvale, CA). For lysosomal compartment tracking, cells were incubated with Alexa fluor 647 BSA (Thermofisher Scientific) for 1 hr followed by washing with HEPES-Saline (135 mM NaCl, 1.2 mM $MgCl_2$, 0.8 mM $MgSO_4$, 28 mM dextrose, 2.5 mM $CaCl_2$, and 25 mM HEPES, pH 7.4) to label endocytic/lysosomal compartments. Incubation with 100nM

Lysotracker Green (Thermofisher Scientific) allow for labeling of acid compartments (lysosome). For tracking of autophagic compartments, cells were transfected with mCherry-GFP-LC3 using lipofectamine. Live cell images were acquired by spinning disk confocal microscopy using a CARV II spinning-disk unit (Crisel Instruments, Rome, Italy) with DAPI, Cy2, Cy3, Cy5 fluorescence filters, an iXon 897 EMCCD camera (Andor Technologies, Belfast, Ireland), and PhotoFluor metal halide white light excitation (Chroma Technologies, Bellows Falls, VT). The system is run with Metamorph Software (Molecular Devices LLC, Sunnyvale, CA), contains on an Olympus iX71 temperature enclosed stand heated to 37ºC, and an automated X-Y-Z stage (Applied Scientific Instrumentation, Eugene, OR) and 60x, 1.4 NA oil lens. *In vitro* assay movies were acquired by wide field multichannel fluorescent microscopy using a DG-4 xenon 175 W excitation lamp (Sutter Instrument Company), a Sedat Quad dichroic mirror (Chroma Technologies), and a Lambda 10–20 emission filter wheel (Sutter Instrument Company, Novato, CA) with filters for DAPI, FITC, Cy3, and Cy5 or equivalent wavelengths and collected on a CoolSNAP HQ cooled CCD camera (Photometrics, Roper Scientific, Tucson AZ). The system was also run with Metamorph Software, an Olympus iX71 temperature enclosed stand heated to 37ºC, and a 60x, 1.4 NA oil lens. In vitro assays were collected at 1 frame per 2.5 sec. Live cell movies were acquired at 1 frame every 2 sec for 82 or 42 sec, with 4-8 wells and 10-15 total fields per condition. Media was changed 5 hours before imaging and controls were performed on the same day for all conditions. Movies were processed in ImageJ (Schneider *et al.* 2012) and Fiji (Schindelin *et al.* 2012) including digital stabilization(Li 2008) and tracking of single cells. Fluorescent spots were automatically tracked using Imaris Software (Bitplane, Belfast, UK) with manual marking of the cell center. The resulting Microsoft Excel files for spot track parameters were compiled using software written for MatLab (Mathworks, Natick, MA), including calculation of track displacement towards the cell periphery (MT-D) using the cell center mark (Schafer *et al.* 2014; Toops *et al.* 2015). Final data and statistics were plotted and calculated in Graphpad Prism.

Immunostaining and image analysis

Indirect immunofluorescence was performed following conventional procedures. Briefly, cells were grown on coverslips, fixed in either cold methanol or 4% paraformaldehyde, blocked and permeabilized (1% BSA, 2% newborn calf serum and 0.01% Triton X-100), and then incubated with the primary and corresponding fluorophore-conjugated secondary antibodies as described previously (Bejarano *et al.* 2014). All slides were mounted for microscopy using Fluoromount-G (SouthernBiotech) containing DAPI (4′,6-diamidino-2-phenylindole) to highlight the nucleus. Images were collected using Axiovert 200 fluorescence microscope (Carl Zeiss) equipped with a ×63 1.4 NA oil objective and ApoTome.2 system. All the images were prepared using Adobe Photoshop 6.0 software (Adobe Systems). For quantitative analysis, a single image was taken at the section of the maximum nucleus diameter and the number of fluorescent cytosolic particles (puncta) per cell and occupied area per total cell area were determined using the Analyze Particles function of ImageJ (NIH) after applying a fixed threshold to all images. All quantifications were done blindly. 3D reconstruction images were modelled as mixed rendering using the Inside4D module for AxioVision Rel. 4.8 after applying the Nyquist sampling criteria. All representative images include quantification in the same or next panel and the number of repetitions is indicated in the corresponding figure legends. For organelle staining, cells were incubated with MitoTracker (50 mM) or Lysotracker (100 nM) (Invitrogen) for 20 min 37°C prior to fixation. In the case of isolated APGs, vesicles were incubated for 10 min at room temperature with primary antibodies, followed by incubation with fluorescence-conjugated secondary antibodies for an additional 10 min as previously described (Koga *et al.* 2011). Labeled vesicles were recovered by centrifugation and spotted on a glass slide, fixed with 8% formaldehyde in 0.25 M sucrose for 15 min, and visualized with a 100× objective and 1.4 numerical aperture in the Axiovert 2000 fluorescence microscope.

Immunoblot and electrophoresis

Subcellular fractions (20 µg of protein/lane) and homogenates (100 µg of protein/lane) from fed or 6-h-starved mice liver were subjected to immunoblot according to conventional procedures (Towbin *et al.* 1979) Briefly, samples were run on SDS–PAGE gels, transferred to nitrocellulose membranes, and, after blockage with low fat-milk, incubated with primary antibodies in 3% BSA. Cell lysates were prepared by solubilization in RIPA buffer (1 % sodium deoxycholate, 0.1 % SDS, 0.15 M NaCl and 0.01 M sodium phosphate, at pH 7.2) containing protease and phosphatase inhibitors. The solubilized fraction was recovered in the supernatant after centrifugation at 12,000 g for 30 min, and protein concentration was measured by the Lowry method using bovine serum albumin (BSA) as a standard (Lowry *et al.* 1951). The proteins of interest were visualized by chemiluminescence using peroxidase-conjugated secondary antibodies in an LAS-3000 Imaging System (Fujifilm, Tokyo,Japan). Densitometric quantification was performed in unsaturated images using ImageJ (National Institutes of Health, Bethesda, MD).

Autophagy assays

Autophagic flux was measured by immunoblot analysis as changes in levels of autophagic markers (LC3-II and p62) upon inhibition of lysosomal proteolysis (Tanida *et al.* 2005). Autophagosome content was evaluated as the number of fluorescent puncta after immunostaining with antibodies against endogenous LC3 (Kabeya *et al.* 2000). Monitorization of the mitochondrial delivery to lysosomes was performed by using the mitophagy reporter mt-Keima as described previously (Katayama *et al.* 2011). Quantification of fluorescence intensity of mt-Keima (Katayama *et al.* 2011) in GFP-channel (FL_{mito}) and Rhodamine-channel (FL_{lyso}) was used to calculate the mitophagy index as ratio of the intensity (FL_{lyso}/FL_{mito}). Ultrastructure analysis by electron microscopy was carried out as descried previously (Bejarano *et al.* 2014). Briefly, culture cells were fixed in 4% paraformaldehyde/0.1% glutaraldehyde in 100mM sodium cacodylate, at pH 7.43, dehydrated and embedded in LR-White resin (LADD Research

Industries). All grids were viewed on a JEOL 100CX II transmission electron microscope at 80 kV (JEOL, Peabody, MA). Morphometric analysis of transmitted electron micrographs was done blinded using ImageJ (NIH). Autophagic vacuoles were identified using standard criteria as previously (Bejarano *et al.* 2014). Briefly, vesicles were catalogued as autophagosomes or autolysosomes if they meet two or more for the following criteria: for autophagosomes they should have a double membrane (completely or partially visible), absence of ribosomes in the outer membrane, luminal density comparable to the surrounding cytosol and identifiable organelles or regions of organelles in their lumen; for autolysosomes they should have similar size but less than 40% of membrane visible as double, luminal density lower than surrounding cytosol and luminal material not recognizable as specific organelles and/or amorphous material. Primary and secondary lysosomes (identified as single membrane vesicles of higher density and smaller average diameter) were excluded from the quantification. The term autophagic vacuole was used for those instances in which differentiation between autophagosomes and autolysosomes was not possible. *In vitro* fusion of autophagosomes and lysosomes in a microtubule-free system was measured as described before (Koga *et al.* 2010). Briefly, isolated APGs or lysosomes were incubated for 10 min at room temperature with primary antibodies against LC3 and LAMP2A, followed by incubation with fluorescence-conjugated secondary antibodies for an additional 10 min. Labeled vesicles were recovered by centrifugation and carefully resuspended in fusion buffer (10 mM HEPES, pH7; 10 mM KCl; 1.5 mM, MgCl2; 1 mM DTT; 0.25 M sucrose; and protease inhibitors) and were mixed in the reaction buffer (3 mM ATP, 2 mM GTP, 2 mM CaCl2, 0.16 mg/ml creatine phosphokinase, 8 mM phosphocreatine, and protease inhibitors) and incubated at 37°C for 30 min. The mixture was spotted on a glass slide, fixed with 8% formaldehyde in 0.25 M of sucrose for 15 min, and visualized under the fluorescence microscope as above.

Additional References

Bejarano E, Yuste A, Patel B, Stout RF, Jr., Spray DC, Cuervo AM (2014). Connexins modulate autophagosome biogenesis. *Nat Cell Biol*. **16**, 401-414.

Kabeya Y, Mizushima N, Ueno T, Yamamoto A, Kirisako T, Noda T, Kominami E, Ohsumi Y, Yoshimori T (2000). LC3, a mammalian homologue of yeast Apg8p, is localized in autophagosome membranes after processing. *EMBO J*. **19**, 5720-5728.

Katayama H, Kogure T, Mizushima N, Yoshimori T, Miyawaki A (2011). A sensitive and quantitative technique for detecting autophagic events based on lysosomal delivery. *Chem Biol*. **18**, 1042-1052.

Kimura S, Noda T, Yoshimori T (2007). Dissection of the autophagosome maturation process by a novel reporter protein, tandem fluorescent-tagged LC3. *Autophagy*. **3**, 452-460.

Koga H, Kaushik S, Cuervo AM (2010). Inhibitory effect of intracellular lipid load on macroautophagy. *Autophagy*. **6**, 825-827.

Koga H, Kaushik S, Cuervo AM (2011). Protein homeostasis and aging: The importance of exquisite quality control. *Ageing Res Rev*. **10**, 205-215.

Li K (2008). Th image stabilizer plugin for ImageJ. http://www.cs.cmu.edu/~kangli/code/Image_Stabilizer.html.

Lowry OH, Rosebrough NJ, Farr AL, Randall RJ (1951). Protein measurement with the Folin phenol reagent. *J Biol Chem*. **193**, 265-275.

Marzella L, Ahlberg J, Glaumann H (1982). Isolation of autophagic vacuoles from rat liver: morphological and biochemical characterization. *J Cell Biol*. **93**, 144-154.

Massey AC, Follenzi A, Kiffin R, Zhang C, Cuervo AM (2008). Early cellular changes after blockage of chaperone-mediated autophagy. *Autophagy*. **4**, 442-456.

Murray JW, Wolkoff AW (2007). In vitro motility system to study the role of motor proteins in receptor-ligand sorting. *Methods Mol Biol*. **392**, 143-158.

Schafer JC, Baetz NW, Lapierre LA, McRae RE, Roland JT, Goldenring JR (2014). Rab11-FIP2 interaction with MYO5B regulates movement of Rab11a-containing recycling vesicles. *Traffic*. **15**, 292-308.

Schindelin J, Arganda-Carreras I, Frise E, Kaynig V, Longair M, Pietzsch T, Preibisch S, Rueden C, Saalfeld S, Schmid B, Tinevez JY, White DJ, Hartenstein V, Eliceiri K, Tomancak P, Cardona A (2012). Fiji: an open-source platform for biological-image analysis. *Nat Methods*. **9**, 676-682.

Schneider CA, Rasband WS, Eliceiri KW (2012). NIH Image to ImageJ: 25 years of image analysis. *Nat Methods*. **9**, 671-675.

Tanida I, Minematsu-Ikeguchi N, Ueno T, Kominami E (2005). Lysosomal Turnover, but Not a Cellular Level, of Endogenous LC3 is a Marker for Autophagy. *Autophagy*. **1**, 84-91.

Toops KA, Tan LX, Jiang Z, Radu RA, Lakkaraju A (2015). Cholesterol-mediated activation of acid sphingomyelinase disrupts autophagy in the retinal pigment epithelium. *Mol Biol Cell*. **26**, 1-14.

Towbin H, Staehelin T, Gordon J (1979). Electrophoretic transfer of proteins from polyacrylamide to nitrocellulose sheets: procedure and some applications. *Proc Natl Acad Sci*. **76**, 4350-4354.

Treichel U, Paietta E, Poralla T, Meyer zum Buschenfelde KH, Stockert RJ (1994). Effects of cytokines on synthesis and function of the hepatic asialoglycoprotein receptor. *J Cell Physiol*. **158**, 527-534.
